# Structural basis of RIP2 activation and signaling

**DOI:** 10.1038/s41467-018-07447-9

**Published:** 2018-11-26

**Authors:** Qin Gong, Ziqi Long, Franklin L. Zhong, Daniel Eng Thiam Teo, Yibo Jin, Zhan Yin, Zhao Zhi Boo, Yaming Zhang, Jiawen Zhang, Renliang Yang, Shashi Bhushan, Bruno Reversade, Zongli Li, Bin Wu

**Affiliations:** 1 School of Biological Sciences, Nanyang Technological University, Singapore, 637551 Singapore; 2 NTU Institute of Structural Biology, Nanyang Technological University, Singapore, 636921 Singapore; 3 Institute of Medical Biology, A*STAR, Singapore, Singapore; 4 Department of Paediatrics, National University of Singapore, Singapore, Singapore; 5 Institute of Molecular and Cellular Biology, A*STAR, Singapore, Singapore; 6 Medical Genetics Department, Koç University School of Medicine (KUSOM), Istanbul, Turkey; 7 Reproductive Biology Laboratory, Academic Medical Center (AMC), Amsterdam-Zuidoost, The Netherlands; 8 Department of Biological Chemistry & Molecular Pharmacology, Howard Hughes Medical Institute, Harvard Medical School, Boston, MA 02115 USA; 9 Present Address: Medical Research Council, University of Cambridge, Cambridge, CB2 0XY UK

## Abstract

Signals arising from bacterial infections are detected by pathogen recognition receptors (PRRs) and are transduced by specialized adapter proteins in mammalian cells. The Receptor-interacting-serine/threonine-protein kinase 2 (RIPK2 or RIP2) is such an adapter protein that is critical for signal propagation of the Nucleotide-binding-oligomerization-domain-containing proteins 1/2 (NOD1 and NOD2). Dysregulation of this signaling pathway leads to defects in bacterial detection and in some cases autoimmune diseases. Here, we show that the Caspase-activation-and-recruitment-domain (CARD) of RIP2 (RIP2-CARD) forms oligomeric structures upon stimulation by either NOD1-CARD or NOD2-2CARD. We reconstitute this complex, termed the RIPosome in vitro and solve the cryo-EM filament structure of the active RIP2-CARD complex at 4.1 Å resolution. The structure suggests potential mechanisms by which CARD domains from NOD1 and NOD2 initiate the oligomerization process of RIP2-CARD. Together with structure guided mutagenesis experiments at the CARD-CARD interfaces, we demonstrate molecular mechanisms how RIP2 is activated and self-propagating such signal.

## Abstract (web summary)

The pathogen recognition receptors NOD1/2 recognize bacterial cell wall components and signal through their downstream adapter kinase RIP2 via a CARD (Caspase activation and recruitment domain) mediated oligomerization process. Here the authors present the cryo-EM structure of the active RIP2-CARD filament and discuss implications for NOD1/2-RIP2 signalling.

## Introduction

Bacterial and viral infections are sensed by their corresponding pathogen recognition receptors (PRRs) in humans. Upon recognizing their substrates, many PRRs propagate the danger signals to downstream adapter proteins through a pyrin/CARD-mediated oligomerization process, which eventually leads to inflammatory responses^1^. Certain adapter proteins, such as mitochondria antiviral signaling protein (MAVS)^2,3^ and apoptosis-associated speck-like protein containing a CARD (ASC)^4,5^ serve as signaling hubs that integrate activation signals from more than one upstream PRR. Dissecting the structural features of the super complexes formed by these adapters is important for a better understanding of how innate immune signaling is regulated^6^.

**Fig. 1:**
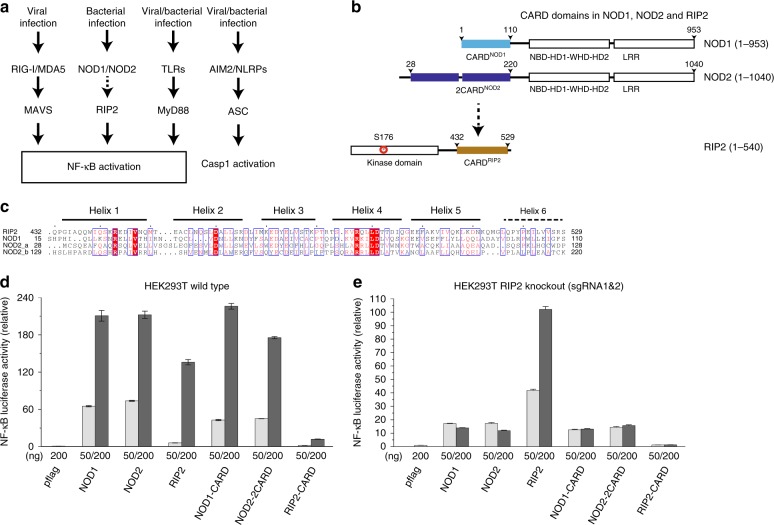
CARD domains from NOD1/2 and RIP2 are important for signaling. **a** Schematic overview of canonical RLR–MAVS, NOD1/2–RIP2, TLR–MyD88, and ASC signaling pathways. Adapters with CARD/pyrin domains relaying the activation signal from PRRs to their corresponding downstream effectors. The dashed arrow indicates previously proposed interactions between NOD1/2 and RIP2 via their CARD domains. **b** Illustrations of protein domain structure of human NOD1, NOD2, and RIP2. The CARD domains are highlighted in colors (light blue for NOD1–CARD, blue for NOD2–CARD, and orange for RIP2–CARD). **c** Sequence alignment of RIP2–CARD, NOD1–CARD, NOD2–CARDa, and NOD2–CARDb. Conserved residues are highlighted in orange and red colors. **d**, **e** NF-κB promoter activation by NOD1, NOD2, RIP2 proteins, and their CARD domains in HEK293T and HEK293T RIP2 KO cell lines. HEK293T cells were transfected with empty pflag vector or NOD1, NOD2, RIP2, RIP2–CARD, NOD1–CARD, and NOD2–CARD together with pGL4.32 NF-κB-RE vector, and CMV-*Renilla* vectors. The amount of plasmids that were transfected was calibrated with pFlag plasmids to ensure that the same amount of plasmids were used in each transfection. Cells were harvested 24 h post transfection and the level of NF-κB promoter activity was measured by dual luciferase assay according to the manufacturer’s instructions. Error bar represents standard deviation values of three independent repeats

NOD1 and NOD2 were among the first PRRs studied and detect conserved bacterial cell wall features (NOD1 detects g-D-glutamyl-meso-diaminopimelic acid (iE-DAP) and NOD2 recognizes muramyl dipeptide (MDP)^7,8^) and signal through their downstream adapter protein RIP2 (a.k.a. RIPK2, CARDIAK, or RICK)^9–14^. RIP2 has a C-terminal CARD domain presumably perceiving the upstream activation signal, and an N-terminal kinase domain that could dimerize and phosphorylate other downstream factors, including TAK1 and NF-κB (Fig. 1a–c)^15,16^. Loss-of-function mutations in NOD1/2 and RIP2 are associated with autoimmune diseases, such as Crohn’s disease and lupus^17–21^. Intriguingly, RIP2 is also involved in NOD1/2-independent signaling events, such as autophagy and neuronal activation^22–25^. Despite previous studies based on computational homology modeling, pull-down, and luciferase reporter assays^26–29^, the molecular basis for RIP2 activation remains incompletely understood. These studies also showed that many mutations of surface residues on the NOD1/2–RIP2–CARD domains were disruptive to their functions. For instance, it remains unclear which of the two NOD2–CARD domains is responsible for RIP2 activation. There are existing efforts using drugs, like ponatinib, to inhibit the RIP2 signaling, however, mostly from the point-of-view of inhibiting kinase activities. Understanding how NOD1 and NOD2 interact with RIP2 would also significantly enhance our understanding of disease-related mutations in these proteins. For example, there are several mutations in NOD1 and NOD2 associated with increased prevalence of inflammatory bowel diseases^30^. Many of these disease-associated mutations, although they do not directly occur within the CARD domains, play important roles in mediating the NOD1/2–RIP2 interactions^17^. An understanding of the molecular mechanisms of RIP2 activation would shed light on how complex innate immune signaling is regulated.

In this study, we first examined human RIP2 complexes that were directly extracted from HEK293T cells and confirmed that they formed filaments upon activation by NOD1/2. Second, we reconstituted homogeneous RIP2–CARD filaments and determined the 4.1-Å resolution cryo-EM structure of the active complex. We then constructed the atomic model of RIP2–CARD filament and validated it using pairwise charge-reversal mutagenesis. These findings provided a direct view of how RIP2–CARD is organized in a C1 left-handed helical filament upon activation. The RIP2–CARD filament structure offers insights into how NOD1–CARD and NOD2–CARD interact with RIP2–CARD. Together with other biochemical evidences, we show that NOD1/2 CARD domains transiently interact with RIP2–CARD, and subsequently induce the oligomerization of this innate immune adapter protein, similar to what has been previously observed in RIG-I/MDA5–MAVS signaling complexes. Last, we engineered fusion protein constructs to capture the possible modes of interactions between NOD1/2 and RIP2 and show that they adapt an unconventional top-down conformation.

## Results

### CARD domains from NOD1/2 and RIP2 are important for signaling

**Fig. 2:**
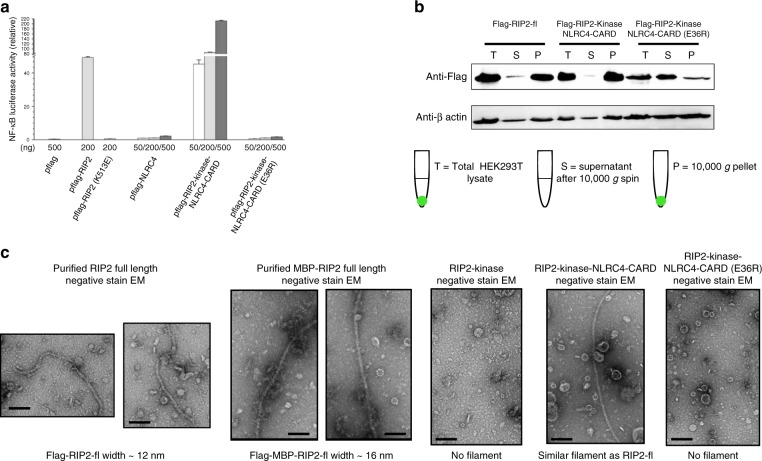
RIP2 forms a filamentous structure upon activation. **a** NF-κB promotor activation by domain swap chimeric constructs of RIP2. HEK293T cells were transfected with empty pflag vector, wild-type RIP2, and chimeric RIP2 together with pGL4.32 NF-κB-RE vector and CMV-*Renilla* vectors. Human NLRC4–CARD (1–100) was used to replace the RIP2–CARD domain (432–540) in human RIP2. Human NLRC4–CARD (E36R) is a monomeric mutant. Error bar represents standard deviation values of three independent repeats. **b** Tendency of oligomerization of wild-type and chimeric RIP2 proteins. HEK293T cells were transfected with flag-tag wild-type RIP2 or chimeric RIP2 constructs. Total cellular extracts (T) were subjected to 10,000 × *g* precipitation for 5 min to isolate the supernatant fraction (S) and the pellet fraction (P). A vast majority of RIP2 appeared to be in the pellet fraction. In NLRC4–CARD swapped constructs, a vast majority remained in pellet form. Chimeric constructs with monomeric NLRC4–CARD (E36R) appeared to be mostly soluble. Western blots were representative of three independent experiments. **c** Negative stain EM images of flag-RIP2, flag-MBP–RIP2, RIP2-kinase, RIP2-kinase-NLRC4–CARD, and RIP2-kinase-NLRC4–CARD (E36R) purified from HEK293T transfected cellular extracts. Flag-RIP2 filaments are about 12-nm thick, similar to RIP2-kinase-NLRC4–CARD. The diameter of flag-MBP–RIP2-fl filaments is wider at 16 nm. No filament was observed for the rest two samples. Scale bar: 100 nm

We first monitored NF-κB transcriptional activities to validate the functional roles of CARD domains in NOD1/2 and RIP2. Overexpression of the full-length proteins and the CARD domain constructs (Fig. 1d and Supplementary Figure 1a) triggered NF-κB activation, as measured with a luciferase reporter assay. RIP2–CARD was less potent at activating NF-κB, probably due to its lack of kinase domain, which may further dimerize and presumably interacts with further downstream factors. High concentration of RIP2–CARD alone is not sufficient. In order to further validate that RIP2 is important for amplifying the NOD1/2 signals, we repeated the experiment after first suppressing intrinsic RIP2 expression using a CRISPR–Cas9-based editing tool, using four independent sgRNAs (Fig. 1e and Supplementary Figure 1b, c). After depletion of the intrinsic RIP2 in HEK293T cells, both NOD1/2 full-length and NOD1/2 CARD domains failed to activate NF-κB, which demonstrates that RIP2 is the predominant downstream signaling adapter for NOD1/2. A functionally retarded mutation within the RIP2–CARD domain (K513E) significantly reduces the NF-κB activity (Fig. 2a). To validate whether the signaling activity of RIP2 is dependent on its CARD domain, we swapped RIP2–CARD with NLRC4–CARD that is known to form filamentous structures^31^. Human NLRC4 alone does not stimulate NF-κB in HEK293T cells, thus, NLRC4 would be a good negative control for domain swap analysis (Fig. 2a). When fused with NLRC4–CARD, RIP2-kinase domain regained strong NF-κB activity. However, signaling was severely disrupted when the NLRC4–CARD contained a disruptive mutation that prevents filament formation (Fig. 2b, c). Thus, we conclude that RIP2 signaling requires a pro-oligomeric CARD domain and that the RIP2-kinase domain alone is insufficient.

### RIP2–CARD oligomerization activates downstream signaling

Crude sedimentation experiments demonstrated that the MAVS oligomer, a classic example of innate immune adapter protein, is the active signaling state^32^, and we performed similar experiments to show that this is also the case for RIP2. After 5 min of 10,000 × *g* centrifugation, activated RIP2 was concentrated in the pellet fraction, similar to MAVS (Fig. 2b). Chimeric RIP2 constructs with NLRC4–CARD domain were also examined by sedimentation and analyzed by anti-FLAG western blotting probing for the N-terminal FLAG-tagged RIP2-kinase domain. Wild-type RIP2 was highly concentrated in pellet fraction, whereas its kinase domain without an oligomer forming CARD domain (NLRC4–CARD with E36R mutation) appeared to be more soluble (Fig. 2b). This observation is in good agreement with luciferase assay results (Fig. 1d). Both ubiquitination and phosphorylation of RIP2-kinase domain are considered as hallmarks of active innate immune signaling^33,34^. When CARD domain constructs of NOD1/2 or RIP2 were expressed, we observed enhanced RIP2 phosphorylation in large molecular complexes, which suggested that the oligomerized RIP2 tends to be correlated with enhanced phosphorylation in the cell (Supplementary Figure 1d). In addition, NOD2–CARD induced oligomerization of RIP2 also correlated with enhanced RIP2 ubiquitination (Supplementary Figure 1e, f). These observations collectively indicate that the induced oligomerization of RIP2 is not an overexpression artifact, but instead is highly correlated to its active signaling capacity in mammalian cells.

### Oligomerized RIP2 forms a filamentous structure

To investigate the structure of the activated oligomeric form of RIP2, full-length FLAG-tagged RIP2 overexpressed in HEK293T cells was purified using anti-FLAG M2 agarose beads, and concentrated for negative stain EM examination. Filamentous objects, about 12 nm in diameter, were identified under the electron microscope, suggesting that active RIP2 might adapt a filamentous helical structure. In order to confirm the identity of these filaments, we repeated the experiment using an MBP-tagged RIP2 construct and identified slightly thicker filaments with a diameter of ~16 nm. This observation correlated well with the expected filament size, since the extra MBP tag containing RIP2 filaments has a wider diameter. In addition, such a filamentous structure was not observed when the FLAG–RIP2-kinase domain alone or a FLAG–RIP2-kinase-NLRC4–CARD construct with a defective mutation E36R was expressed (Fig. 2c). Direct examination of the MBP–RIP2 filaments obtained from HEK293T extracts provided further information on the overall morphology and size of the complex. However, due to the heterogeneity of the sample and insufficient sample density, we were unable to determine the cryo-EM structure of the mammalian-expressed RIP2 full-length constructs. Only vague structural information, like the apparent pitch distance between two protruding points could be measured.

**Fig. 3:**
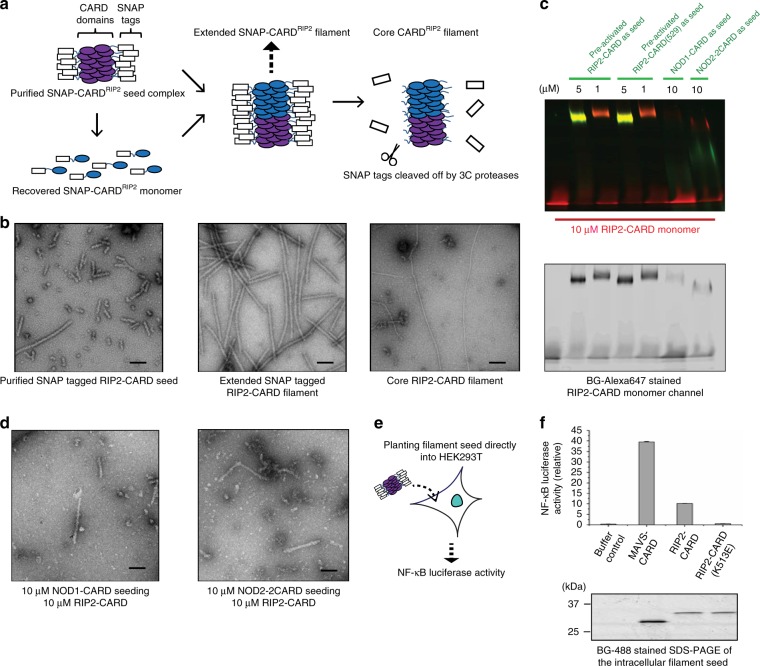
Preparation of recombinant RIP2–CARD filament in its signaling active form. **a** Cartoon illustrations showing how recombinant RIP2–CARD filaments were prepared. The oligomeric seed fraction of SNAP–RIP2–CARD was purified and refolded into functional monomers at resting state. Extended filaments were prepared by coincubating monomers and seeds together. Once extended filaments formed, 3C proteases were used to remove the peripheral SNAP fusion tags. The ordered core RIP2–CARD filaments were revealed afterward. **b** Negative stain EM images of SNAP–RIP2–CARD seeds (left panel), extended SNAP–RIP2–CARD filaments (middle panel), and the core RIP2–CARD filaments (right panel) at 49,000× magnification. The core RIP2–CARD filaments were much thinner (~7 nm) compared to SNAP–RIP2–CARD filaments (~11 nm). **c** Dual-color stained Bis-Tris native gel demonstrating how different seeds, including SNAP–RIP2–CARD (1 or 5 μM), SNAP–NOD1–CARD (10 μM), and SNAP–NOD2–CARD (10 μM), colored in green, triggered the induced oligomerization process of SNAP–RIP2–CARD (10 μM) monomers, colored in red. Monomer channel was shown in the lower panel in black and white. **d** Negative stain EM images showed that 10 μM RIP2–CARD proteins were induced to form filaments by 10 μM NOD1–CARD or 10 μM NOD2–CARD. Scale bar: 100 nm. **e** Cartoon illustration of NF-κB promoter luciferase assay with SNAP–RIP2–CARD protein seed directly electroporated into the cells. **f** NF-κB was activated by intracellular recombinant RIP2–CARD seeds. SNAP–RIP2–CARD seeds, SNAP–RIP2–CARD (K513E) seeds, and MAVS–CARD–SNAP were electroporated into HEK293T cells, together with pGL4.32 NF-κB-RE vector and CMV-*Renilla* vectors. Only oligomeric RIP2 protein constructs led to activation of NF-κB, but not the K513E, the monomeric form of RIP2. MAVS–CARD–SNAP was used as a strong positive control. The same amount of proteins were transfected in each sample. BG-Alexa 488 dye stained SDS-PAGE conferring that the similar level of recombinant seeds were catapulted into the reporter cells. Error bar represents standard deviation values of three independent repeats

To obtain more detailed structural information of active RIP2–CARD filaments, a reconstitution assay was developed to recapitulate the RIP2 activation process in vitro. Based on the intrinsic molecular architecture of RIP2, the N-terminal kinase domain was swapped with a protein fusion, SNAP-tag, to improve the protein stability during chemical refolding (Fig. S1a). These recombinant SNAP–RIP2–CARD seeds (pre-activated oligomer) and monomers (resting state) were prepared using a slightly modified protocol from previous MAVS studies^35–37^. A gentler guanidinium gradient was required to minimize misfolding and precipitation. The resting state RIP2–CARD monomer remained monomeric for days when not exposed to any stimulus. When the seed SNAP–RIP2–CARD and the refolded monomeric form were co-incubated, extended SNAP–RIP2–CARD filaments with various lengths were generated, depending on the seed-to-monomer ratio (Fig. 3a, b). This assay was iteratively optimized until most monomers were active and were incorporated into the extended filaments. These reconstituted long filaments were homogeneous and compact (Fig. 3b, middle panel). Both the seed and monomer RIP2–CARD shared the same secondary structure, which is predominantly α-helix, as determined by circular dichroism spectrum analysis (Supplementary Figure 2a). The SNAP protein residing at the filament periphery was then removed to reveal the rigid core composed of RIP2–CARD domains (Fig. 3b, right panel). Wild-type SNAP–RIP2–CARD seeds readily recruited refolded SNAP–RIP2–CARD monomers to their ends and formed long filaments. This growth of filaments was also observed with native gel EMSA (Fig. 3c, Supplementary Figure 2b). Long filaments migrate much slower than smaller fragments on native gels. Besides pre-oligomerized RIP2–CARD itself, both recombinant NOD1–CARD–SNAP and NOD2–CARD–SNAP stimulated RIP2–CARD filament formation, which directly demonstrates their ability to trigger innate immune activation. Interestingly, the newly formed SNAP–RIP2–CARD filaments did not co-migrate with the NOD1/2 seeds as the RIP2–CARD seeds did, suggesting that such an interaction could be highly transient under these conditions (Supplementary Figure 2b), and the activation caught-in-action complex might be unstable. Direct EM examinations of the RIP2–CARD filament stimulated by NOD1–CARD and NOD2–CARD revealed that the formed RIP2–CARD filament was short and more heterogeneous, which is in agreement with its faster migration rate on native gel (Fig. 3d). In brief, the reconstituted RIP2 activation assay confirmed that CARD domains from NOD1 and NOD2 can activate RIP2 by promoting RIP2–CARD oligomerization without the involvement of any additional cofactors and post-translational modifications.

In order to demonstrate that the reconstituted SNAP–RIP2–CARD filament represents the endogenous active state of RIP2, we electroporated recombinant SNAP–RIP2–CARD filaments into HEK293T cells (Fig. 3e). This experiment confirmed that recombinant RIP2 oligomeric seeds can directly trigger the NF-κB luciferase signal in the cell (Fig. 3f). Only oligomeric wild-type SNAP–RIP2–CARD fragments and the positive control of recombinant MAVS–CARD seeds, were able to activate NF-κB efficiently, while the monomeric mutant SNAP–RIP2–CARD(K513E) was unable to do so (Fig. 2f). These findings demonstrate that in vitro-reconstituted RIP2–CARD filament can directly initiate intrinsic cellular signaling in a filamentous structure-dependent manner.

### Molecular structure of the RIP2–CARD filament

**Fig. 4:**
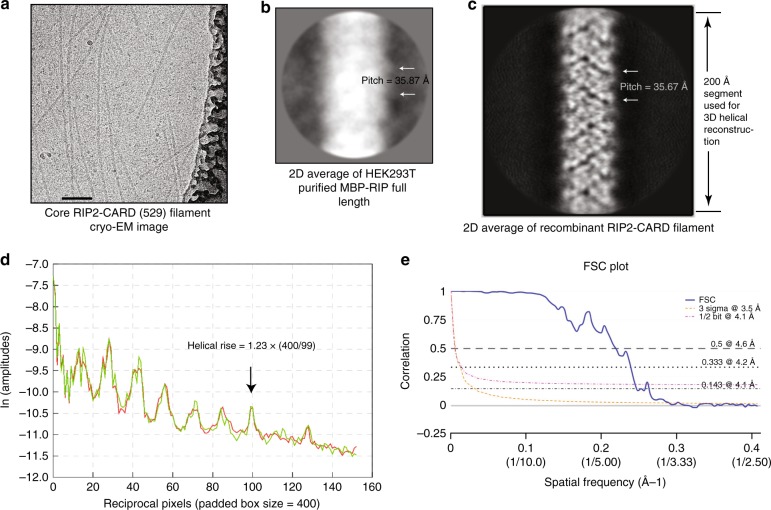
Electron density of RIP2–CARD filament resolved using helical reconstruction. **a** Cryo-EM micrograph of core RIP2–CARD–EM construct. Scale bar: 100 nm. **b** Representative 2D class average of MBP–RIP2 extracted from HEK293T cells. About 500 segments were used to generate this preliminary 2D average class. By manual counting of the pixels between the two protrusions along the filament. Visual helical pitch was found to be about 35.87 Å. **c** 2D class image of the core RIP2–CARD–EM generated when picking 140,000 segments. Visual helical pitch was found to be about 35.67 Å. **d** The helical layer line profile from Relion analysis of the RIP2–CARD filament data. The last signal peak (from left to right) indicates the position of the last (furthest) layer line, which could be used to calculate helical rise parameter. **e** The FSC plot showed that the resolution for the overall density of the entire segment is 4.1 Å, at FSC = 0.143 gold standard

The homogeneity of the in vitro-reconstituted RIP2–CARD filaments allowed the structure determination of this filament. Long and straight core RIP2–CARD filaments were generated by optimization of the CARD domain boundaries. A 13 amino acid tag was added to the N-terminal of residue 432 of RIP2–CARD and the last 11 amino acids at the C-terminal end of RIP2–CARD were removed to stabilize the core RIP2–CARD filament and prevent filament bundling on the EM grid. This construct is called RIP2–CARD–EM. After removal of the SNAP fusion tags, the core RIP2–CARD–EM filaments were further purified by size-exclusion chromatography and then vitrified onto EM grids and imaged as described in previous studies (Fig. 4a)^35^. Using the helical reconstruction module in Relion 2.0, we picked and sectioned the filaments and got more than 600k particles. After iterative rounds of 2D classification, dozens of 2D classes with good density and power spectrum features started to emerge. We selected the particles from the best 2D classes for further 3D refinement. Since the 2D average images of MBP–RIP2 from the full-length construct expressed in HEK293T cells were of very low quality (Fig. 4b), we relied on the pitch size to tell whether our recombinant RIP2–CARD filaments resemble the structural features of full-length RIP2. Here, the pitch is the distance between the repeated protruding points of the RIP2 filament. On the negative stain EM image (Fig. 4b), this distance is 17 pixels (2.11 Å per pixel), which corresponds to 35.87 Å. On the cryo-EM image (Fig. 4c), this distance is 29 pixels (1.23 Å per pixel), which is 35.67 Å. In the final solved 3D structure, the measured distance between the two protrusion points on a 2D projection is (720/101.36)*4.96 = 35.23 Å. The three numbers are close, which is a strong indication that our structural interpretation of the density is correct, and that the RIP2–CARD oligomerization state reflects that of full-length RIP2. In order to determine the helical symmetry parameters, we conducted power spectrum analysis as suggested by Relion 2.0 manual^38^, as well as instructions from previous publications^39^, to narrow down the range (Fig. 4d). Based on further analysis using SPRING^40^, the helical rise was calculated to be within the range of 4.9–5.1 Å, and the helical rotation was around −101 or +101°. Further 3D refinement and post processing of the segment density were performed using Relion 2.0, and confirmed the left-hand helicity (−101°) based on the density map. In order to obtain a better assignment of the central segment density, we cropped out density representing 12 centrally located RIP2–CARD monomers using Chimera and conducted pseudo-crystallographic refinement using Phenix real space refinement. The resulting molecular model provides useful structural information until 3.5~4.1Å, with a helical rise equal to 4.936 Å and rotation equal to −101.4° (Fig. 4e, Supplementary Figure 3a–c and Supplementary Table 1).

**Fig. 5:**
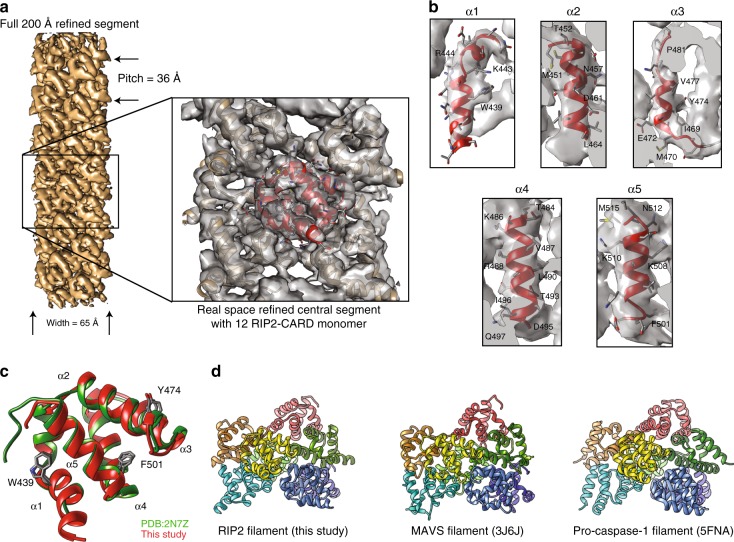
A RIP2–CARD 12mer structure based on cryo-EM density. **a** Cartoon illustration of the average segment density of activated RIP2–CARD filamentous structure (EMDB code: 6482). The image was prepared using Chimera with auto-adjusted contour level. Twelve monomers of RIP2–CARD were fitted into the central segment according to the protocol. **b** Close view of the electron density. Five different α-helices from RIP2–CARD fitted nicely into the density map, with the aromatic side chains at important references for determining the registry. **c** Comparison of RIP2–CARD monomer models from PDB (green) and this study (red). **d** Different filamentous structures composed of CARD-containing proteins including RIP2, MAVS, and pro-caspase-1. It was quite apparent to notice their shared helical symmetry

The refined RIP2–CARD filament was a left-handed C1 helical filament (Fig. 5a), and was stabilized by three classic types of interfaces—Type I, II, and III interfaces seen in death-fold super family proteins^41^. We were able to unambiguously assign most of the amino acids with bulky side chains (Fig. 5b). When we overlapped our monomer structure with a previous monomer solution NMR structure (PDB:2N7Z)^42^, we observed small differences in the overall domain architecture (Fig. 5c and Fig. S3d). This was similar to other death domain oligomers and little conformational changes were observed between monomeric and oligomeric states. However, we need to perform real space refinement to obtain reasonable side chain conformations and establish surface interactions in all three interfaces (Supplementary Figure 3e, f). By cross-comparing some of the known CARD domain oligomers (Fig. 5d), we found that the helical symmetry for RIP2–CARD filament, MAVS–CARD filament, and pro-caspase-1 CARD filament^43^ was almost identical. All of these filaments have a helical rise of around 5 Å, and a helical rotation of around −101°. This could be a coincidence with all adapting a geometry favored by the globular CARD domain, or this observation might hint that potential structure crosstalk was possible among these innate immune players. Further investigations are needed to test this hypothesis.

**Fig. 6:**
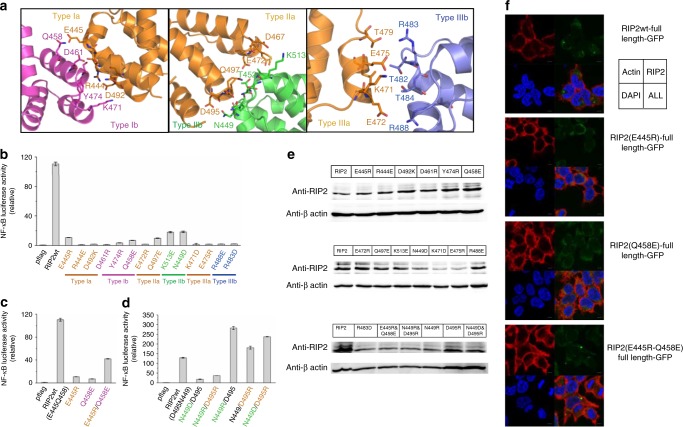
Interdomain surface interactions within the RIP2–CARD filament. **a** Cartoons illustrated the details of Ia:Ib, IIa:IIb, and IIIa:IIIb interactions in RIP2–CARD filament. Residues at the interface are shown in stick models. **b** NF-κB promoter activation by RIP2 and RIP2 single-point mutants. These RIP2 point mutations localized on the interaction interfaces and showed loss of function in terms of NF-κB promoter activation. **c** NF-κB promoter activation by RIP2, RIP2 single-point mutants E445R, R458E, and RIP2 double mutant E445R andR458E. The single mutants all showed loss of ability to activate NF-κB promoter, while the E445R and R458E double mutant partially restored NF-κB promoter activation. Error bar represents standard deviation values of three independent repeats. **d** NF-κB promoter activation by RIP2 and RIP2 mutants in HEK293T cells. RIP2 single-point mutants N449D and RIP2 double mutant N449R andD495R showed loss of function. While RIP2 single-point mutants N449R, D495R, and double mutant N449D and D495R showed higher NF-κB luciferase activities. Error bar represents standard deviation values of three independent repeats. **e** Expression levels of all RIP2 mutants are similar. HEK293T cells were transfected with wild-type RIP2 and RIP2 mutants. Cells were harvested 24 h post transfection. Levels of over-expressed wild-type RIP2 and RIP2 mutants were measured by anti-RIP2 antibody. Western blots were representative of three independent experiments. **f** Confocal microscope images of wild-type RIP2–GFP, RIP2(E445R)–GFP, RIP2(R458E)–GFP, and the double mutant RIP2(E445R–R458E)–GFP. The charge-reversal double mutant regained the puncta formation activity. Gray scale bar: 5 μm

As shown in Fig. 6a, Type I (orange and red) and Type II (orange and blue) interfaces contributed most to the interdomain interactions and several pairs of charged interactions were identified. Due to the flexible nature of the surface interactions, one residue could potentially interact with multiple corresponding binding partners from the other surface. We were not able to map out all the individual interaction pairs, but we identified residues that are important for stabilizing the oligomeric state. As a typical Type I interface, residues from helix 1 and 4 (orange, Type Ia, R444, E445, and D492) interact with charged side chains from helix 2 and 3 (red, Type Ib, Q458, D461, Y474, and K471). Consistent with previous studies^26,27,29^, we also identified the same group of residues that play important roles in stabilizing the RIP2–CARD oligomer. R444, D492, and D461 were proposed by the Monie group to be important for RIP2–CARD interactions with NOD1 and RIP2 itself^27^. Y474 sits at an important interface mediating interaction between Type Ia and Ib, which explains why a tyrosine-to-phenylanine mutation would disrupt RIP2 activity^44^. In Type II interactions, residues at the turns between helix 2/3 and helix 4/5 (orange, Type IIa, D495, Q497, E472, and D467) mainly interact with residues located within the turn between helix 1/2 (green, Type IIb, N449, T452, and K513). Type III (orange and blue) interface was relatively weak and it was stabilized by interactions between residues from helix 2 (orange, Type IIIa, T479, E475, K471, and E472) and coiled turn between helix 3/4 (blue, Type IIIb, R483, T482, T484, and R488). Identification of these residues was not surprising because many of them were shown to disrupt RIP2 signaling before^26,27^, including E472, E475, R483, and R488. However, here we propose a detailed oligomeric RIP2–CARD model based on a direct density observation. Overall, the stability of the RIP2–CARD filament was maintained by a collection of weak charged interactions involving almost all surfaces of RIP2–CARD. Since CARD domains are globular in shape, predicting their oligomeric interactions is extremely challenging, since a few degrees of rotation would result in a completely different set of residues on their surfaces. Our cryo-EM structure will facilitate further studies of this signaling complex.

To validate the relevance of the solved filament structure in cellular innate immune signaling, point mutations of critical surface residues were introduced in full-length RIP2 constructs and the functional impact of these mutations was assessed using NF-κB luciferase assays. As expected, most of the single mutations that disrupt important surface interactions between RIP2–CARD monomers showed reduced cellular activity (Fig. 6b).

To further confirm the structural model and to validate the exact interaction pairs, pairwise charge-reversal mutagenesis was done. Cellular activities were completely abolished when we disrupted the native interaction pairs E445–Q458 and N449–D495 (Fig. 6c–e). When the interactions at these sites were rescued by introducing reversely charged interactions pairs, E445R–Q458E double mutant regained its activity and was six times more active than any of the single mutants. When repelling residues like Arg–Arg or Asp–Asp pairs were introduced at the N449–D495 positions, RIP2 became inactive. Intriguingly, when the original elastic N449–D495 interaction pair was replaced by stronger charge–charge pairs, like N449R–D495 and N449–D495R, the mutants were more active than the wild-type protein, demonstrating how the oligomeric tendency of RIP2 governs its cellular roles. These luciferase assay data supported our structural model and provided insights into the plasticity of CARD surface interactions. The flexible pairing of surface residues serves as the key of the molecular imprinting message, as long as the topology of template lock matches. In addition, we went forward to put the single and charge-reversal mutants into the GFP-tagged mammalian expression constructs. Under the confocal microscope, we observed that both RIP2 formed puncta-like structures, while the E445R and Q458E individual mutations disrupted the puncta formation (Fig. 6f). This further supported our structure model of RIP2–CARD oligomer.

### Both NOD1 and 2 activate RIP2 in a top-down interaction mode

Both NOD1–CARD and NOD2–CARD alone without the participation of any other cofactors were sufficient to induce the oligomerization of RIP2–CARD in our reconstituted assays (Supplementary Figure 4a). Upon mixing with NOD1/2, RIP2–CARD starts to oligomerize, while there is no obvious co-migration of the NOD1/2 seeds together with the oligomerized RIP2–CARD. We conducted additional EM experiments to analyze whether NOD1/2 might form a stable complex with stimulated RIP2–CARD. There is evidence suggesting that such complex formation is possible, but it is transient and unstable, when compared to seeding activities using oligomerized RIP2–CARD, as shown in Supplementary Fig. 4b, c. In addition, the induced oligomerization activity is not due to crowding effects. We conducted induced oligomerization experiments using two different monomers, RIP2 and NLRC4–CARD monomers. Only their corresponding seeds induced their oligomerization, but not any other seed complexes, as shown in Supplementary Figure 4d.

**Fig. 7:**
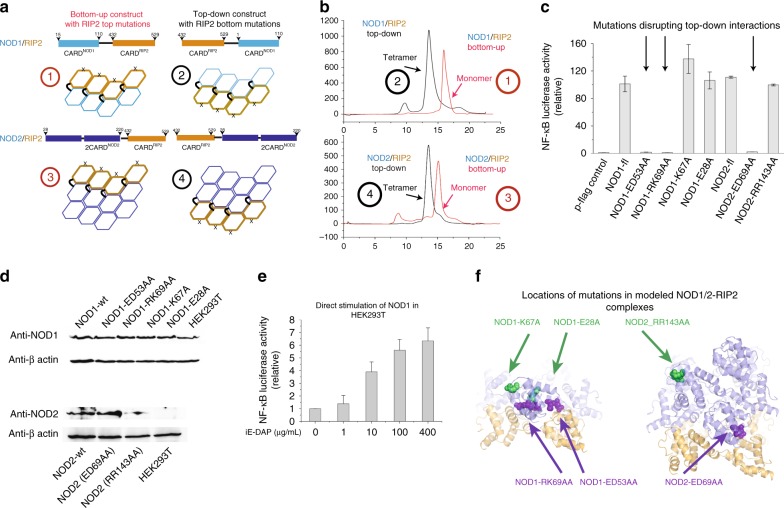
Both NOD1 and 2 activate RIP2 in a top-down interaction mode. **a** The design of NOD1/RIP2 and NOD2/RIP2 fusion constructs. According to our structural model, we introduced mutations located on RIP2–CARD surfaces to prevent aggregation. **b** Size-exclusion chromatographic profiles of bottom-up and top-down constructs of NOD1/RIP2 and NOD2/RIP2 fusions. Both top-down constructs (constructs 1 and 3 in Fig. 6a) appeared to form stable tetramers in solution, not the other way around. Since RIP2–CARD tends to self-oligomerize, N449A–K513E mutant of RIP2–CARD domain was used to prevent self-interactions in bottom-up constructs, and R444E–E445R mutant of RIP2–CARD domain was used in top-down constructs. Without incorporating the RIP2–CARD mutations, fusion constructs precipitate. **c** NF-κB promoter activation by NOD1, NOD2, NOD1 mutants, and NOD2 mutants in HEK293T cells. HEK293T cells were transfected with empty vector, wild-type NOD1, wild-type NOD2, NOD1 mutants, and NOD2 mutants together with pGL4.32 NF-κB-RE vector and CMV-*Renilla* vectors. Cells were harvested 24 h post transfection and the level of NF-κB promoter activity was measured by dual luciferase assay according to the manufacturer’s instructions. **d** Expression levels of NOD1, NOD2 constructs, and their mutants were similar. HEK293T cells were transfected with wild-type NOD1/2 as well as their mutants. Endogenous levels of NOD1 and NOD2 in HEK293T cells were also monitored. Western blots were representative of three independent experiments. **e** NF-κB promoter activation after iE-DAP stimulation in HEK293T cells. Error bar represents standard deviation values of three independent repeats. **f** Illustrations highlighting the locations of the NOD1/NOD2 mutations tested in (**a**). Green arrows highlighted mutations on the top surfaces of NOD1/2 CARD domains, while purple arrows highlighted the bottom mutations. Only bottom surfaces from NOD1/2 CARD domains were functionally important. RIP2 utilizes the same interface to interact with both NOD1 and NOD2

Our next goal was to identify how NOD1/2 interacts with RIP2. Primary sequences of CARD domains are poorly conserved, which makes the prediction of the mode of potential interactions difficult. Actually, it is still not fully understood whether it is the first CARD or the second CARD in NOD2 that interacts with RIP2–CARD. In order to identify the mode of interactions between NOD1/2–RIP2, i.e., top-down, bottom-up, or bidirectional, we engineered two sets of fusion protein (Fig. 7a). The first set has C-terminal (up position) RIP2–CARD with either NOD1–CARD or NOD2–CARD at the N-terminal (bottom position). The second set of constructs was reversed in their domain architecture. To eliminate unwanted self-aggregation of RIP2–CARD, selective top or bottom surface mutations on RIP2–CARD were introduced, based on our filament structure. N495A–K513E double mutants were introduced to disable the top surfaces of RIP2–CARD in unwanted self-oligomerization, and R444E–E445R mutants were used to silence the RIP2–CARD bottom surfaces. These fusion constructs behaved well during protein expression and purification. However, their size-exclusion profiles appeared to be drastically different (Fig. 7b). Both top-down constructs exhibited a migration profile close to ~120 kDa, which was about four times of their monomer sizes, and the bottom-up constructs appeared to be mostly homogeneous monomers (~30 kDa). This suggested that top-down tetramer complexes of fusion proteins were more stable, and both NOD1 and NOD2 likely activate RIP2 in a top-down manner, through cooperative and transient interactions with the same RIP2–CARD top surface, composed of Type Ib and IIb interfaces. Cellular luciferase assays of NOD1 and NOD2 full-length constructs with either top or bottom-silenced CARD were also conducted (Fig. 7c, d). Consistent with the previous observations that K67 in NOD1–CARD did not affect the NOD1–RIP2 interaction, while R69 and K70 were critical for the interaction^27^, as well as the hypothesis that NOD2–CARDa was responsible for direct RIP2 interaction^26^. We noticed in previous investigations of NOD2–CARD surfaces, several residues on the first CARD (CARDa) were found important for the NOD2–RIP2 interactions. Interestingly, during our western-blot analysis, we found that there is no detectable NOD2 expression in HEK293T (Fig. 7d). Direct NOD1 stimulation using iE-DAP yields a mild luciferase response (Fig. 7e), which indicates the presence of endogenous NOD1 in HEK293T, although not as high as the induced level. Mutations on the top surfaces of NOD1 and NOD2 did not affect the downstream signaling, while the mutations on the bottom surfaces severely abrogated their activities (Fig. 7f). This observation is in agreement with our models based on the biochemical analysis.

## Discussion

As an important antibacterial defense mechanism, NOD1, NOD2, and RIP2 have been intensely studied for years^26,27,30^. However, previous investigations did not fully dissect the signaling mechanism of the oligomeric signaling complexes formed by these proteins on a molecular level. Our in vitro reconstitution approach overcame the biggest hurdle of dissecting heterogeneous signaling complexes since it enabled us to prepare homogeneous filamentous samples that are suitable for structural investigations. Our structure reveals the interdomain interactions that mediate RIP2–CARD filament formation.

Filamentous structures are not uncommon in cellular immune signaling. Several other innate immune adapter proteins form higher-order complexes with helical symmetries, like MyD88 in TLR signaling pathways, MAVS in RLR signaling pathways, and ASC in canonical inflammasome complexes. Interestingly, these independent innate immune seeding complexes composed of DD super family domains (RLR/MAVS, NOD1/2/RIP2, and MyD88/IRAK4)^35,45^ share striking similarities among them. An ASC–CARD filament was also proposed to have the same helical symmetry^46^. The helical symmetry parameters of these complexes are almost the same (Fig. 5d). Beyond innate immunity, DD super family proteins are abundant and mediate versatile signaling events that are also critical for apoptosis, necrosis, pyroptosis, autophagy, and other life-or-death decisions^47,48^. It would be intriguing to investigate whether the potentially shared overall helical symmetry among death domain plays complicated functional roles.

In this study, we demonstrated that the innate immune adapter RIP2 forms a helical oligomeric structure upon activation. Our findings contribute toward the understanding of how DD super family proteins communicate with each other to facilitate cellular signaling.

Our biochemical and cell-based activity data also show that the bottom surface of the NOD1–CARD and NOD2–CARDa are the responsible interaction surfaces mediating oligomerization of RIP2. This interaction resembles those seen in RIG-I/MDA5–MAVS signaling pathways^49^: in both MAVS and RIP2, their activators interact with these adapter proteins in a transient way. Under conditions of which little or no stable complexes were observed in native gel or size-exclusion chromatography, oligomerization of the downstream adapter proteins could be induced efficiently. In native intracellular signaling environment, cofactors (K63-polyubiquitin and/or virus-induced oligomerization of RIG-I/MDA5, bacterial substrates-induced oligomerization of NOD1/2, etc.) could enhance the stability of the seeding complex, hence influencing innate immune activity.

We used high concentrations of NOD1/2 monomers in our in vitro assays to bypass the requirement of cofactors. Further studies are needed to identify potential cofactors and post-translational modifications of NOD1/2–RIP2–CARD domains, which would provide further insights into how these signaling pathways are regulated. Our reconstituted assays reported in this study could serve as a clean platform testing various regulatory mechanisms. The RIP2–CARD filament structure could also help to interpret disease-associated mutations in NOD1/2 and RIP2.

## Methods

### Plasmid construction

To prepare plasmids for mammalian cell expression, wild type and variants of NOD1 full-length (1–953, synthesized by Creative Biogene), NOD2 full-length (1–1040, synthesized by Creative Biogene), and flag-tag RIP2 full-length (1–540, cDNA from MegaMan Human Transcriptome Libraries) were cloned into pcDNA3.1 between the HindIII and XhoI restriction sites (Supplementary Tables 2 and 3). For recombinant CARD proteins, NOD1–CARD (residues 1–110)–SNAP and NOD2–CARD (residues 28–220)–SNAP, RIP2–CARD (residues 432–540)–SNAP were inserted into pcDNA3.1 by HindIII and XbaI restricition sites. Site-directed mutagenesis was performed using the KAPA Hifi PCR kits (KAPA Biosystems).

To generate the CARD–SNAP recombinant proteins, SNAP-tag was inserted between the EcoRI and XhoI restriction sites into pET47b (Novagen). The RIP2–CARD, NOD1–CARD, and NOD2–CARD were then inserted between the XmaI and EcoRI restriction sites. Several SNAP–RIP2–CARD constructs were designed to obtain single filaments instead of bundles for further structural analysis. N-terminal 13 extra amino acids—VDEALREAQTKSA—named 13aa was attached to the N-terminal of RIP2–CARD and then this insert was cloned into the pSnap-tag (T7)-2 vector (New England Biolabs) between the EcoRI and NotI restriction sites after the SNAP sequence. The RIP2–CARD (432–540) domain was further optimized by removing the flexible C-terminal tail to prepare RIP2–CARD (432–529), which was used to collect good-quality RIP2 filament micrographs.

### Protein purification

For the expression of the His-tagged recombinant proteins, the constructed plasmids were transformed into BL21(DE3) (NEB) and grown at 37 °C for 16–18 h. Then the starting culture was transferred to a larger volume of lysogeny broth (LB), which was grown until an OD600 of about 0.6–0.8, then the temperature was lowered to 16 °C, and cells were induced with 0.5 mM IPTG and grown overnight. Bacteria were harvested at 4600 × *g* (Avanti JXN series, Beckman Coulter) for 10 min and then resuspended with lysis buffer (20 mM Tris-HCl, pH 8.0, 300 mM NaCl, 20 mM imidazole, and 10% glycerol). Cells were then lysed by Emulsiflex-C3, and centrifuged at a speed of 30,000 × *g* (Avanti JXN series, Beckman Coulter) for 20 min at 4 °C to separate supernatant and pellet. The supernatant was passed through a pre-equilibrated column containing Ni-NTA agarose beads (ThermoFisher Scientific) by gravity. The column was then washed with lysis buffer containing 20 mM imidazole to remove nonspecific binding proteins. His-tagged proteins were eluted with elution buffer that contained 300 mM imidazole. To further purify our recombinant proteins, size-exclusion chromatography (Superdex 200 increase, GE Healthcare) was performed in buffer A (20 mM Tris-HCl, pH 8.0, 150 mM NaCl, and 1 mM DTT). At this step, the purified proteins were the oligomeric pre-activated seed fraction of RIP2–CARD. For monomeric RIP2–CARD, it was prepared following the protocol as described here. Concentrated and purified soluble pre-activated RIP2–CARD at around 10 mg/ml was buffer exchanged into 10 ml of 6 M guanidinium, and gradually dialyzed against 3, 2, 1.5, 1, 0.8, and 0.6 M guanidinium containing buffer A in the cold room, and eventually in buffer A. In the absence of stimuli, refolded SNAP–RIP2–CARD protein remained monomeric for 24 h.

### Oligomerization assay

RIP2–CARD monomers that were labeled with Alexa Fluor 647 (New England Biolabs) and seeds were incubated with Alexa Fluor 488 (New England Biolabs) for 10 min separately. One microliter of 100 μM BG-Alexa dye stocks (dissolved in water containing 5% DMSO) were added into every 100 μl of pre-labeling protein stock at a concentration of 20 μM. To check whether NOD1–CARD and NOD2-CARD seeds can interact with RIP2–CARD monomer, fluorescently labeled monomeric SNAP–RIP2–CARD (10 μM) was mixed with 5 or 10 μM NOD1–CARD–SNAP and NOD2–CARD–SNAP for 15 min at room temperature before analysis by Bis-Tris native PAGE gel (Life) or 15% SDS-PAGE gel. The double fluorescently labeled gel was run at a voltage of 200 V for 60 min. Once the EMSA was completed, they were visualized by Typhoon FLA7000 (GE Healthcare) with 100-μm pixel size at channels 488 and 647 using PTM 600. To form RIP2 prion-like long filament (200–500 nM), ~20 μM monomeric SNAP–RIP2–CARD was mixed with unlabeled, wild-type SNAP–RIP2–CARD seeds at a molar ratio of 10:1 for overnight. Later on, in-house-prepared MBP-3C-protease^36^ was added to the fully extended filament with a final concentration of 0.5 μM. The digestion was performed at 4° for 8 h, before further purification to separate the core filament, the cleaved-off SNAP tags, and the proteases. Extended SNAP–RIP2–CARD thick filament and 3C-protease-digested core RIP2–CARD thin filament were then purified using size-exclusion column Superose 6 (GE Healthcare), before structural analysis.

### Genome-editing vectors and reagents

All sgRNA-expression vectors were built on the pSpCas9(BB)-2A-Puro (PX459) V2.0 backbone (Addgene plasmid no. 62988). sgRNAs were designed with the GUIDES web-based CRISPR design tool (http://guides.sanjanalab.org/#/). Guide sequences are provided as below. As required, DNA sequences for the guides were modified at position 1 to encode a G, owing to the transcription-initiation requirement of the human U6 promoter.

### CRISPR–Cas9-driven targeted mutagenesis of RIPK2

HEK293T (Sigma-Aldrich) cells were transiently transfected with Lipofectamine 2000 (Invitrogen) as per the manufacturer’s recommendations. As demonstrated by Agudelo et al.^50^, 1 μM ouabain (Sigma) was added directly to the cells during the culture-medium change at day 4 after the transfection, replenished every 3 or 4 days, and maintained for 10 days. Surviving clones of genome-edited HEK293T cells in ATP1A1 and RIPK2 were subsequently used for the downstream experiments.

Sequences for SpCas9 guides

ATP1A1 G2: GATCCAAGCTGCTACAGAAG

RIPK2 G1: GATAATGTATAGTGTGTCACA

RIPK2 G2: GACTATTTTCATGGAGCTGAG

RIPK2 G3: GAGATCATACGTGCTCGGTG

RIPK2 G4: GAGTTTCCTGCAGTTGAGAG

### Luciferase assays

HEK293T cells were plated into 24-well plates (Greiner) 24 h prior to transfection. Plasmid mixtures were pre-mixed with Lipofectamine 2000 (Invitrogen) as per the manufacturer’s recommendations before being added to the cell culture. The media containing transfection reagent was exchanged back to normal media 8 h post transfection. Dual luciferase assays were carried out with the dual luciferase reporter assay system (Promega). Cells from each well were harvested 24 h post transfection and lysed using 200 μl of 1x passive lysis buffer (Promega). Fifty microliters of the cell lysates were aliquoted onto 96-well white flat-bottom chimney well plates (Greiner). Luciferase Assay Reagent II (Promega) was first added to each well and the plate was read in a Tecan plate reader to record luminescence in each sample. Then Stop and Glow® reagent (Promega) was added to each well to quench firefly luciferase activity while allowing measurement of luminescence from *Renilla* luciferase by Tecan plate reader.

The dual luciferase system is consisted of two different reporter genes, firefly (*Photinus pyralis*) and *Renilla* (*Renilla reniformis*) luciferase on two separate plasmids. The gene encoding firefly luciferase is under the control of NF-κB promoter and the gene encoding *Renilla* promoter is fused to a constitutive CMV promoter. The two plasmids were cotransfected into each sample with a ratio of 5:1 for NF-κB-firefly vector:CMV-*Renilla* co-reporter vector combinations. Twenty-four hours post transfection, the cells were harvested and the activities of luciferases were measured sequentially from a single sample.

The relative light units were first calculated as the ratio of firefly to *Renilla* luciferase activity. Then the data were presented as relative light units of induced cells/relative light units of control cells. The blank control samples in each experiment were cells mock-transfected with empty pcDNA3-FLAG vectors. For each plot, the data represented the means of three independent assays with the bars indicating standard error in each sample.

### Tissue culture and transfection

All cells were maintained at 37 °C with 5% CO_2_ in DMEM (Gibco) supplemented with 10% (v/v) fetal bovine serum (Gibco). HEK293T cells were plated at a density of 5 × 10^5^ cells per well for poly-L-lysine- (Sigma-Aldrich) coated six-well plate or 1 × 10^5^ cells per well for a 24-well plate 24 h prior to transfections. Plasmids were transfected into HEK293T cells using FuGENE HD (Promega) with a “5:1“ ratio according to the manufacturer’s instructions.

For protein electroporation, HEK293T cells were at 80% confluency before experiments. Cells were harvested and washed twice with PBS. For each reaction, 2 × 10^6^ cells were resuspended in 100 μL of resuspension buffer R (Thermofisher). Proteins (20 μg) were then transfected using NEON transfection system (Thermofisher) according to the manufacturer’s recommendations. Cells were harvested 8 h post electroporation.

### Immunoblots

HEK293T cells were plated into six-well plates (Corning) 24 h prior to transfection. Cells were harvested 24 h post transfection and lysed using RIPA buffer (150 mM NaCl, 50 mM Tris, pH 8, 1 mM EDTA, 1% (v/v) Triton X-100, 0.1% (w/v) sodium dodecyl sulfate, and 0.5% (w/v) sodium deoxycholate) on ice for 30 min with vortexing every 5 min. Cell lysates were centrifuged for 5 min at 10,000× *g* at 4 °C to separate the pellet and supernatant fractions, and each component was subjected to SDS-PAGE. Actin control western blot was performed using the 18,000 × *g* supernatant fractions of each cell lysate. Transblotting was done by the Mini Trans-Blot® Cell (Bio-Rad) onto PVDF membrane (Bio-Rad), which was then blocked by 10% (w/v) blocking grade powder (Bio-Rad) diluted in TBST (50 mM Tris-HCl, pH 7.4, 140 mM NaCl, and 0.1% Tween20). Next, the blot was incubated with primary antibodies in 1x TBST with 5% (w/v) BSA (Biowest) at room temperature for 1 h or 4 °C overnight. Secondary antibody incubation was performed for 1 h at room temperature. WesternBright Sirius, a femtogram HRP substrate (Advansta) was added to each blot and left for 1 min. The blot was visualized with Chemi-Doc (Bio-Rad).

All the western blots were representative of at least three independent experiments. Lot of information of commercial antibodies was included in Supplementary Table 4. In the displayed figures, anti-RIP2 antibody was used in a (1:500) dilution ratio; anti-phospho-RIP2 antibody was used in a (1:1000) dilution ratio; anti-flag antibody (M2) was used in a (1:10,000) dilution ratio; anti-NOD1 antibody was used in a (1:1000) dilution ratio; anti-rabbit HRP-conjugated secondary antibody was used in a (1:5000) dilution ratio; anti-NOD2 antibody was used in a (1:200) dilution ratio; anti-β-actin antibody was used in a (1:4000) dilution ratio; anti-mouse IgG secondary antibody was used in a (1:5000) dilution ratio; anti-Ub antibody was used in a (1:10,000) dilution ratio.

### Immunoprecipitation

HEK cells transfected with pcDNA3 full-length RIP2 and NOD2 were harvested and lysed using passive 1x RIPA buffer (150 mM NaCl, 50 mM Tris, pH 8, 1 mM EDTA, 1% (v/v) Triton X-100, 0.1% (w/v) sodium dodecyl sulfate, and 0.5% (w/v) sodium deoxycholate). The whole-cell lysate was then incubated with anti-FLAG nanoparticles (Nvigen) at room temperature for 1 h. The beads were collected with magnets and washed three times with wash buffer (100 mM Tris-HCl, pH 8.0, 150 mM NaCl, and 1% Triton X-100). The proteins were eluted with 1.5 mg/mL 3X FLAG® Peptide (Sigma-Aldrich) containing wash buffer.

### Immunofluorescence

293T cells were seeded at ∼70% confluence on poly-L-lysine-coated coverslips. All cells were grown in complete media and allowed to adhere overnight. Coverslips were fixed with 3.7% paraformaldehyde in 1x PBS and permeablized with 0.5% Triton X-100 in 1x PBS. Coverslips were incubated with Alexa Fluor™ 647 Phalloidin (Invitrogen) diluted in PBS with 1% BSA for 30 min at room temperature. DAPI staining was then performed on the coverslips at room temperature for 5 min. Coverslips were mounted with VECTASHIELD HardSet Antifade Mounting Medium (Vector Laboratories).

### Cryo-electron microcopy

For cryo-EM grid preparation, Quantifoil R2/2 holey carbon grid (Quantifoil) was glow-discharged and plunge-frozen using a Vitrobot (FEI) to be treated with 5-µl sample at a concentration of about 3 mg/ml. Final high-resolution images were collected at Tecnai Polara, 300 kV, using SerialEM^51^ for data collection. Cs = 2.0 mm. Gatan K2 camera (Gatan), super-resolution mode for data collection, and binned 2× when doing motion correction. The sub-frame time is 200 ns and total exposure time is 7 s, this will give 35 frames per stack. The pixel size on the final image is 1.23 Å. The dose rate is 8 e/pixel/s.

For cryo-EM data, super-resolution image stacks were gain normalized and binned by 2× to a pixel size of 1.23 Å prior to drift and local movement correction using MotionCorr2^52^. Date processing software Relion 2 was used for CTF determination, particle picking, 2D classification, 3D classification, and refinement procedures. Totally, 3100 micrographs were collected and analyzed. We manually picked out ~18,000 filaments and windowed out segments of 220 × 220 pixels, yielding 653,110 particles for 2D classification. After several iterations, bad particles were removed and we used 236,877 particles for 3D classification. Ab initio low-resolution helical structure was generated using a Gaussian cylinder as an initial model and good 2D class averages, which employed exhaustive searches for orientations of segments that are strictly constrained by the known helical symmetry. This reconstruction served as an initial reference model for 3D classification and auto-refinement in Relion. Finally, 142,230 particles were chosen to calculate our RIP2 filamentous model. All 3D refinements were carried out following the gold-standard procedure where the data set was divided into two half-sets. After refinement was converged, a mask was calculated and applied to the final data set and the corrected Fourier shell correlation (FSC) was calculated to estimate the resolution about 4.1 Å by using FSC = 0.143 criterion. In the final model, parameters of the RIP2 filament were calculated to yield a 4.936 Å of the axial rise per asymmetric unit and an azimuthal rotation per subunit of −101,373°.

### Model building

Step 1 was the monomer docking. The RIP2–CARD monomer structure (PDB accession code: 2N7Z) was used at this stage. The residue numbers were renumbered and the terminal residues were deleted to match the length of the density. The individual helix was manually shifted (rigid body movement) according to the locations of three large aromatic side chains. These three residues (W439, Y474, and F501) are highlighted in Fig. 5b. The density map at this resolution enabled us to unambiguously locate these residues. This step was done in Coot.

In Step 2, a tetramer containing the Type I, II, and III interfaces (a centrally located monomer and three other monomers forming Type I, II, and III interfaces with the central one) was built. After building four monomers in this way, we obtained a tetramer that could be used to refine the surface interactions. Phenix real space refinement was done without rigid body refinement. Different parameters were tested, a torsion-rotational angle NCS constraint (Phenix default auto setting) was used at this step. In this way, all surfaces of the central monomer could be refined.

During Step 3, a tetramer model was built that is in agreement with the data from the cellular activity assays, given particular emphasis in charge-reversal experiments.

In Step 4, a 12mer model was built. The central monomer from Step 3 was copy-and-pasted to occupy the entire 60 Å segment density. In the end, 12 identical monomers were added to the density. Phenix real space refinement with default NCS torsion-rotational constraint was performed. After several rounds of refinement, a model with reasonable statistics was obtained.

Step 5: final refinement. The clash score was >16 after Step 4 and when inspecting this model in detail, some interactions were rebuilt and then Step 4 was repeated until satisfactory statistics and density interpretation were achieved.

## Electronic supplementary material

Supplementary Information

Reporting Summary

## Data Availability

The cryo-EM map has been deposited in the Electron Microscopy Databank with accession code EMD-6482. The atomic coordinate model has been deposited in the Protein Data Bank (PDB) with accession code [5YRN]. All relevant data are available from the corresponding author upon reasonable request. A reporting summary for this article is available as a Supplementary Information file.
