# Supplementary material for: Structural basis of RIP2 activation and signaling
Source: Nat Commun. 2018 Nov 26;9:4993. doi: 10.1038/s41467-018-07447-9 (PMC6255760; doi:10.1038/s41467-018-07447-9)
Supplement: Supplementary file 1 — Supplementary Information [file 41467_2018_7447_MOESM1_ESM.pdf]

Supplementary Information

Qin Gong, Ziqi Long et al. **Structural Basis of RIP2 Activation and Signaling**

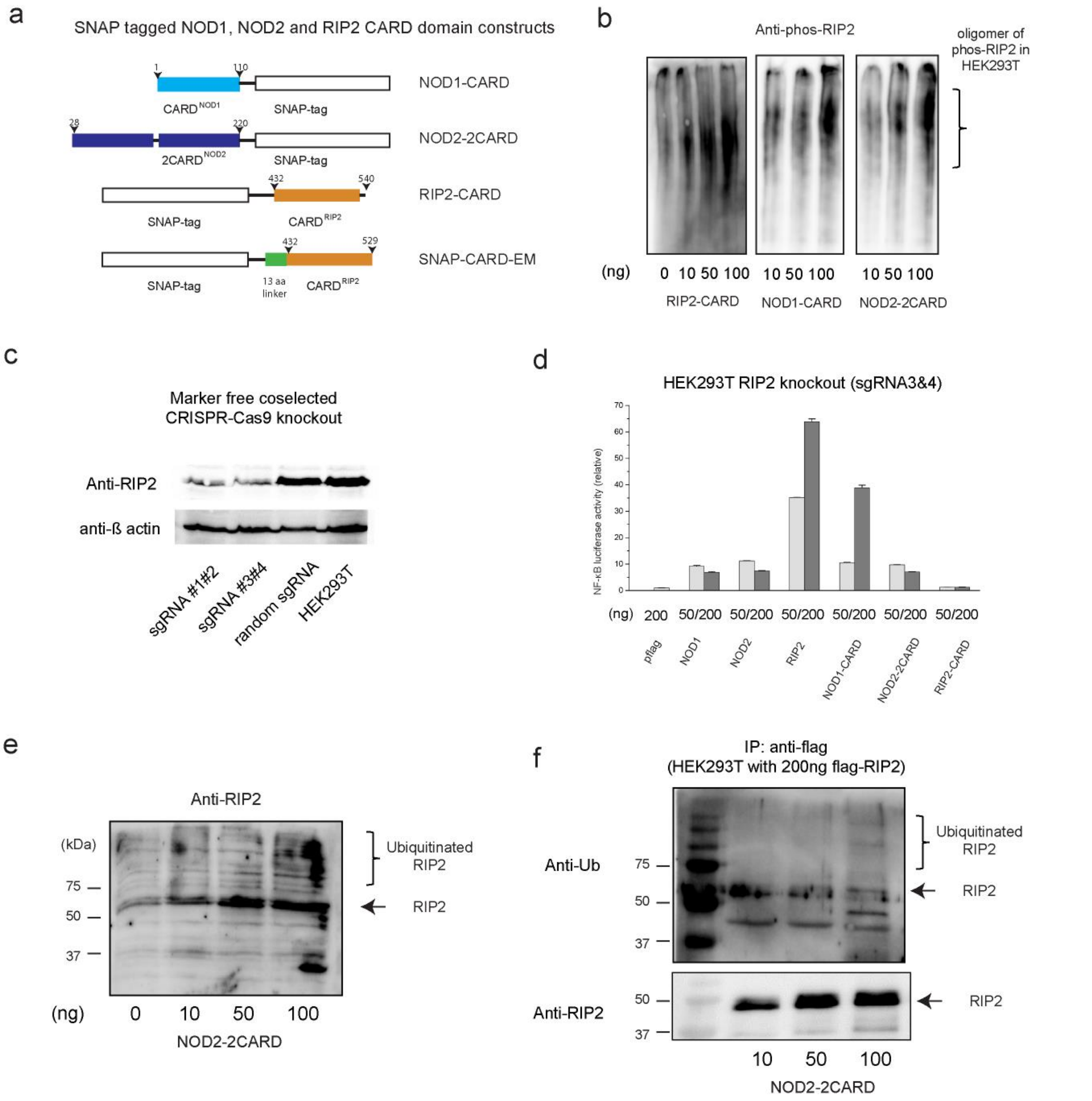

Supplementary Figure 1

(a) Schematic illustration of recombinant CARD domain constructs. SNAP tags were fused to the C-terminal of NOD1-CARD and NOD2-2CARD to replace the rest of the domains. SNAP tag was at the N-terminal of RIP2-CARD to better represent the domain architecture and replace the kinase domain. There is a 3C protease cleavage site in the linker between the CARD domains and SNAP. In SNAP-RIP2-CARD-EM construct, 13 amino acids ‘VDEALREAQTKSA’ were added in front of the CARD domain to stabilize the end, and C-terminal boundary of the RIP2-CARD was truncated to residue 529 to remove the flexible non-structured end.

(b) RIP2 level decreased significantly in HEK293T RIP2 KO cell lines. Levels of endogenous RIP2 were measured with anti-RIP2 antibody in two HEK293T RIP2 KO cell lines, a HEK293T RIP2 KO control cell line and wild-type HEK293T cell line.

(c) NF-κB promoter activation by NOD1, NOD2 RIP2 proteins and their CARD domains. HEK 293T RIP2 KO (sgRNA 3&4) cells were transfected with empty vector or NOD1, NOD2, RIP2, RIP2-CARD, NOD1-CARD, NOD2-2CARD together with pGL4.32 NF-κB-RE vector and CMV-*Renilla* vectors. The amount of plasmids that were transfected was calibrated with pFlag plasmids to ensure same amount of plasmids were transfection in each sample. Cells were harvested 24 hr post-transfection and the level of NF-κB promoter activity was measured by dual luciferase assay according to manufacturer's instructions. Western blots were representative of 3 independent experiments.

(d) Increased RIP2 phosphorylation and oligomerization level induced by larger amount of CARD constructs. HEK293T cells were transfected with an increased amount of CARD domain constructs and the total amount of plasmids used for transfection of each sample was calibrated with empty vector. Cells were harvested 24 hr post transfection and analyzed by anti-phospho-RIP2 western blot. Cellular fractions were loaded onto Bis-Tris native gel to separate molecular complexes based on size and charges. Increased level of oligomerized phosphorylated RIP2 was detected when HEK293T cells more CARD proteins. Error bar represents standard deviation values of three independent repeats.

(e) Increased number of higher molecular bands were induced by an increased amount of NOD2-2CARD-SNAP. HEK293T cells were transfected with an increased amount of NOD2-2CARD-SNAP constructs and the total amount of plasmids used for transfection for each sample was calibrated with empty vector. Cells were harvested 24 hr post transfection and levels of endogenous RIP2 in each sample were measured by anti-RIP2 antibody. Several bands with higher molecular weight were detected on the blot. Western blots were representative of 3 independent experiments.

(f) Increased ubiquitinated RIP2 level was induced by an increased amount of NOD2-2CARD-SNAP. HEK293T cells were transfected with an increased amount of NOD2-2CARD-SNAP constructs and same amount of Flag-RIP2 constructs and the total amount of plasmids used for transfection for each sample was calibrated with empty vector. Cells were harvested 24 hr post transfection and RIP2 was harvest by anti-Flag immunoprecipitation. Levels of RIP2 ubiquitination and RIP2 in each sample were measured by anti-ubiquitin and anti-RIP2 antibody. Western blots were representative of 3 independent experiments.

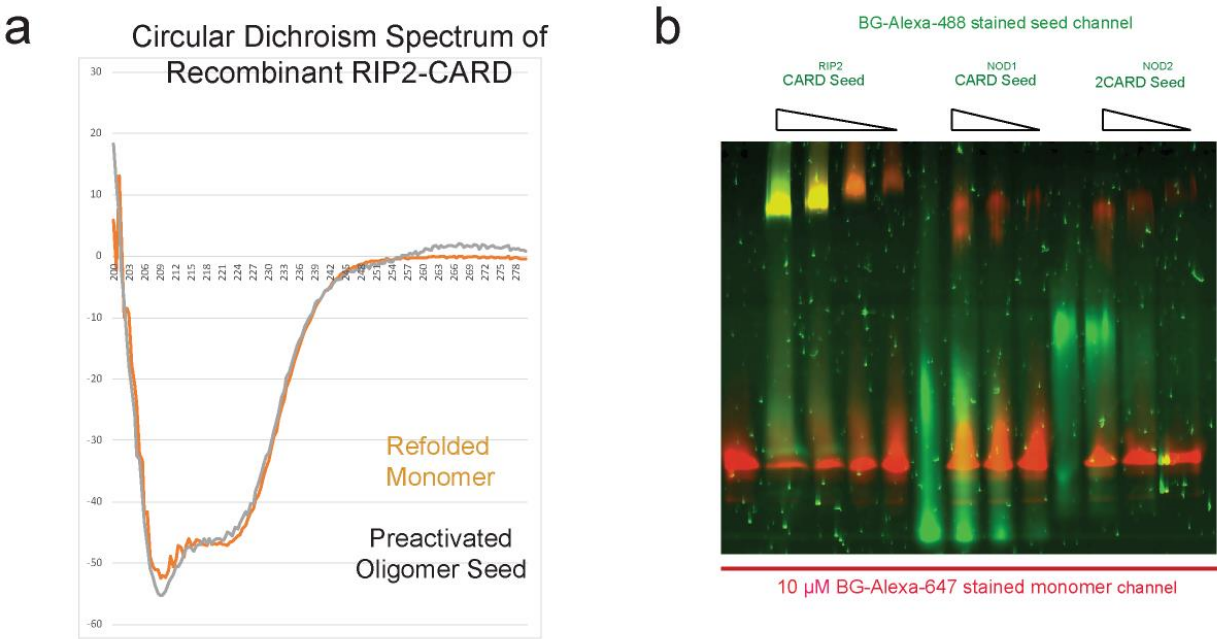

Supplementary Figure 2

(a) Refolded monomer (yellow) and oligomer seed (black) were examined by Circular Dichroism spectrum. They shared similar curves and contained mostly α-helices.

(b) EMSA assays demonstrated how of RIP2-CARD monomers (red) were induced to oligomerization by different seeds, including RIP2-CARD (10, 5, 2, 1 µM), NOD1-CARD (10, 5, 2 µM) and NOD2-2CARD (10, 5, 2 µM), shown in green fluorescence channel.

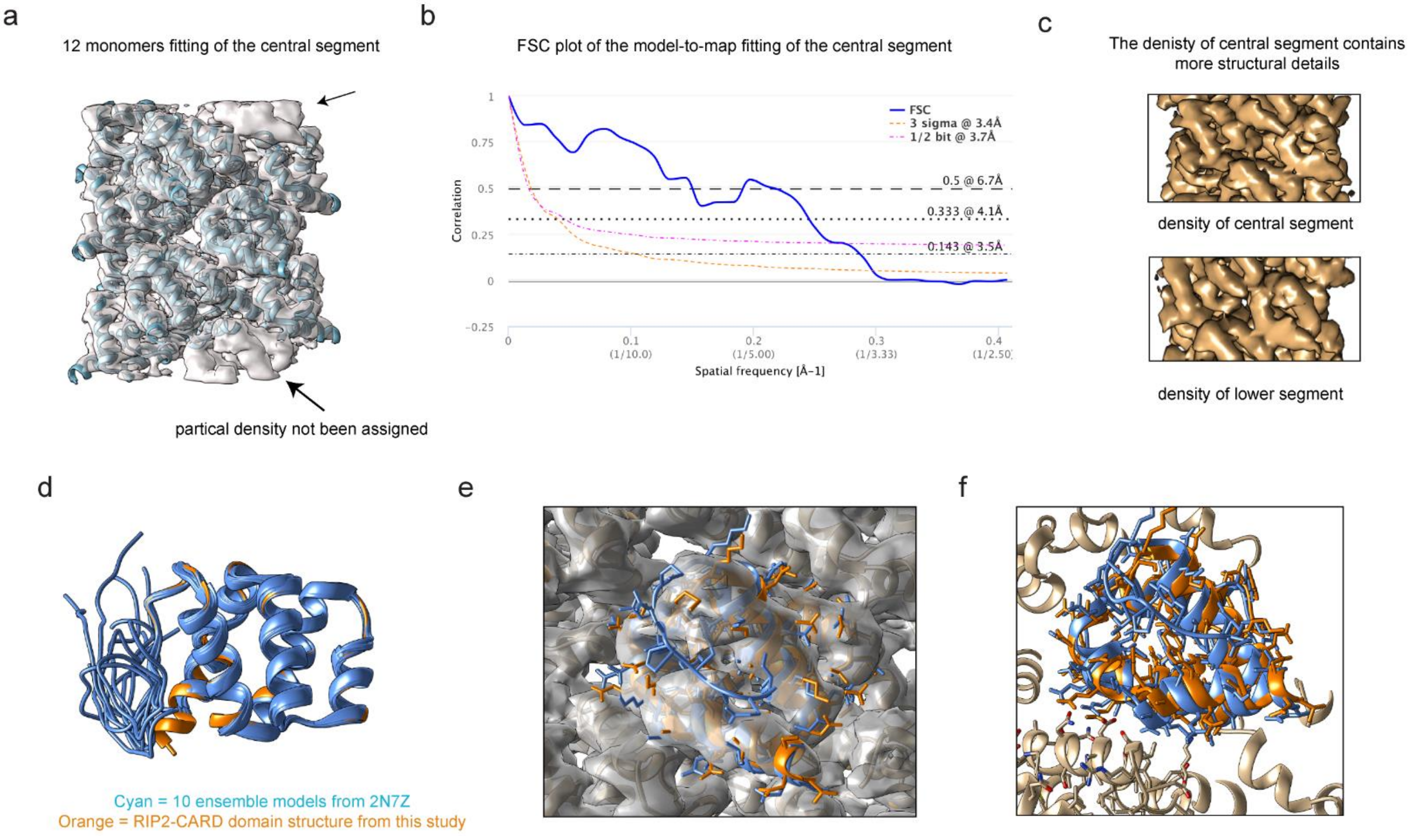

Supplementary Figure 3

(a) The final RIP2-CARD 12mer model (PDB:5YRN) fit to the central segment of the cryo-EM density. There are chunks of density not assigned to any amino acids during pseudo-crystallographic refinement.

(b) Map-to-model FSC plot of the central segment. FSC=0.143 when the resolution cutoff was 3.5 Å.

(c) Schematic view of the central segment density as well as another section slightly away from the center. RELION refined filament density has non-uniform density features along the z-axis.

(d) Overlapped view of 10 conformational states from 2N7Z with the J chain of 5YRN as the reference.

(e) Monomer structure from 2N7Z is not suitable for direct interpretation of the cryo-EM density, due to disordered amino acids towards the ends.

(f) Side chains from 2N7Z monomers are not compatible with interdomain interfaces. Local refinement based on cryo-EM density is necessary to build the RIP2-CARD oligomer model.

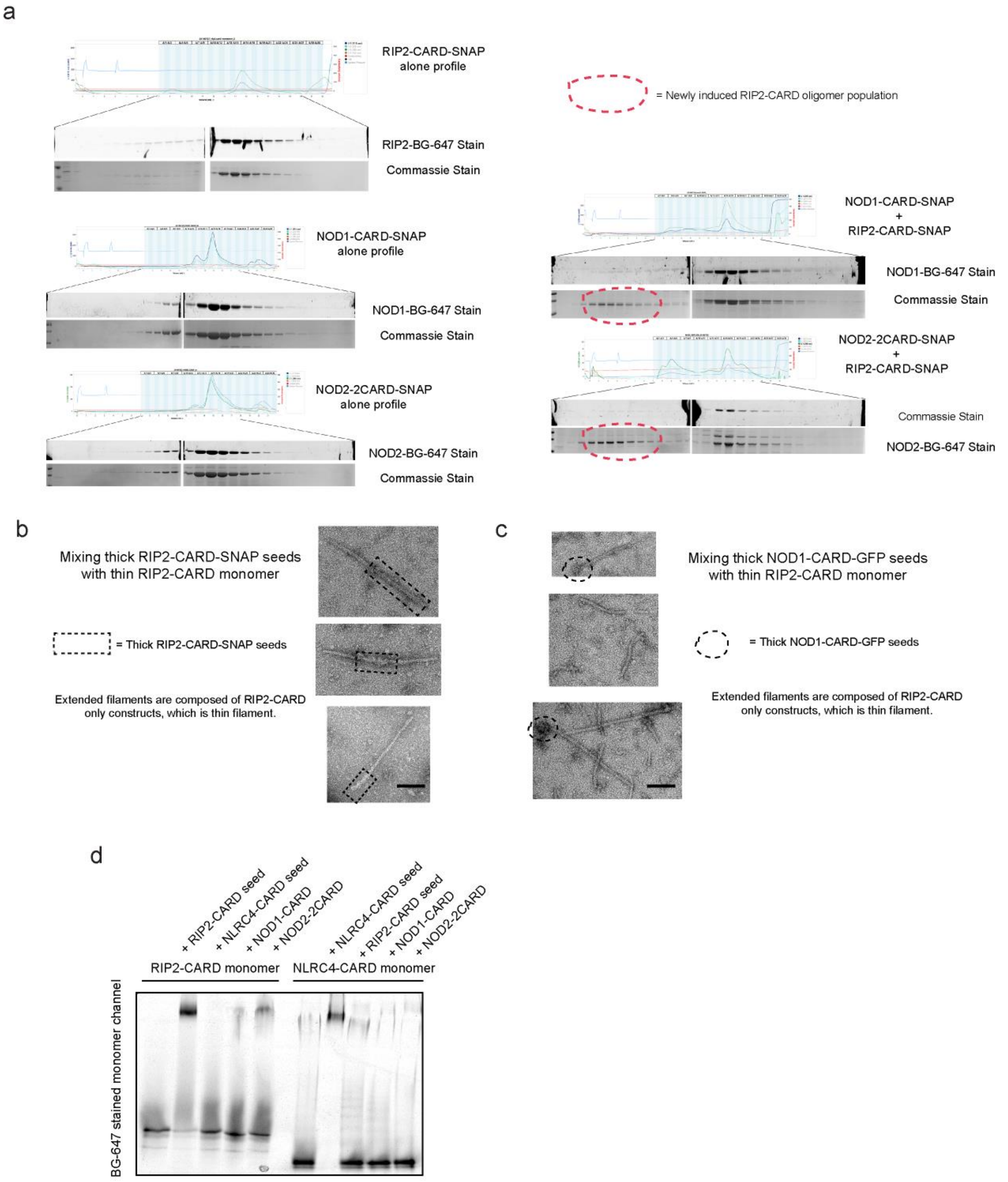

Supplementary Figure 4

(a) Size exclusion chromatographic analysis and SDS-PAGE results of NOD1-CARD, NOD2-2CARD, RIP2-CARD, and the mixture of NOD1-CARD/RIP2-CARD, and mixture of NOD2-2CARD/RIP2-CARD. 600 µl of 20 µM protein solutions dissolved in PBS buffer was injected into 10/300 superdex 200 column. The running profile was recorded using Bio-Rad NGC system.

(b) Negative stain EM images of thin RIP2-CARD filaments. Thicker diameter of RIP2-CARD-SNAP were used to indicate the location of the seeds. RIP2-CARD-SNAP seeds (highlighted by dash boxes) formed stable complex at the ends of the newly extended thin RIP2-CARD filaments. Scale bar: 100 nm

(c) Negative stain EM images of thin RIP2-CARD filaments seeded by NOD1-CARD-GFP. Thicker diameter of NOD1-CARD-GFP (highlighted in dash circles) indicated the location of the seeds. NOD1-CARD-GFP seeds formed less stable complex with the newly extended thin RIP2-CARD filaments. Scale bar: 100 nm

(d) CARD-CARD interaction induced oligomerization requires specific pairing of the interaction partners. When coincubating 10 µM RIP2-CARD or NLRC4-CARD monomers with different seed complexes, only their corresponding functionally relevant seeds (NOD1/2 and RIP2 for RIP2, NLRC4 for NLRC4) could efficiently stimulate the oligomerization of the monomers. NLRC4 failed to activate RIP2, and *vice versa*. 5 µM of different seed complexes were used.

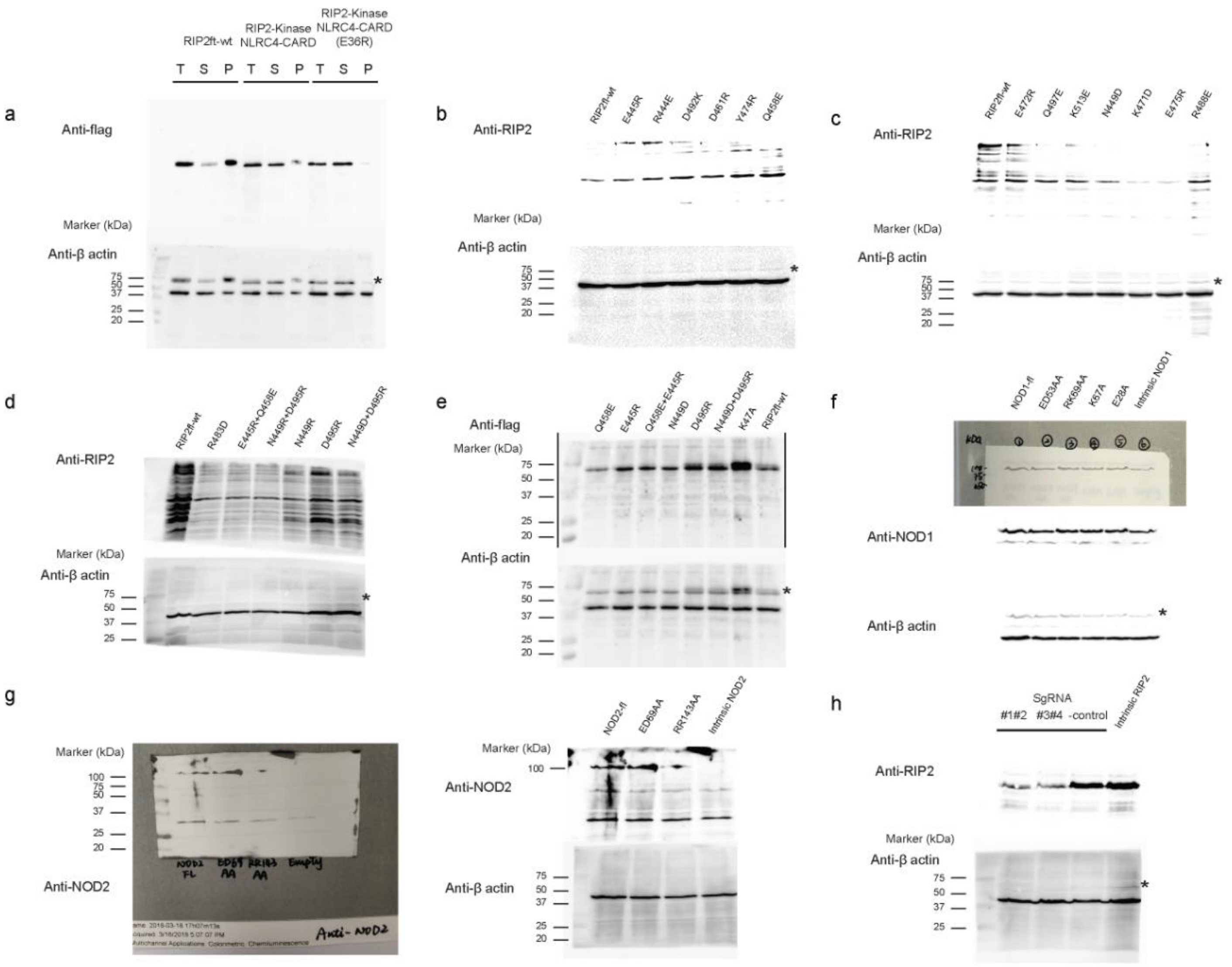

Supplementary Figure 5 Uncropped western blot raw images

- (a) Uncropped data for Figure 2b, * indicates anti-actin blotting could pick up some signal from the other blotting channel.
- (b) Uncropped data for Figure 6e
- (c) Uncropped data for Figure 6e
- (d) Uncropped data for Figure 6e
- (e) Additional anti-flag validation of crucial charge reversal RIP2-CARD mutants
- (f) Uncropped data for Figure 7d
- (g) Uncropped data for Figure 7d
- (h) Uncropped data for Supplementary Figure 1

Supplement Table 1. Data Collection, map and model refinement, validation

**Data Collection**	
Microscope	Tecnai Polara
Voltage (kV)	300
Detector	K2
Pixel size (Å)	1.23
Defocus range (μm)	-2.0
Electron Dose (e^-/A^2)	8
**Helical Reconstruction**	
Software	RELION 2.0
Segment length (Å)	200
Particles	142,230
Helical rise (Å)	4.936
Helical rotation (°)	-101.373
Resolution (Å)	4.1
**Coordinate Refinement (mid-segment 12mer)**	
Software	Phenix
Unit Cell Dimension (Å)	76.26 x 76.26 x 76.26
Resolution Cut Off	3.5
Rwork	0.4415
Rfree	0.4545
**Model**	
Number of residues	1044
B-factor overall	202.7
R.M.S. deviation	
Bond length (Å)	0.007
Bond angle (°)	0.994
**Validation**	
Molprobity clashscore (percentile)	8.18
Rotamer Outliers (%)	0
C_β deviation (%)	0
CC* central segment density	0.6988
CC* model (5YRN) to map	0.8083
CC* (3.9 – 3.5 Å)	0.1982
Ramachandran plot	
Fovored (%)	95.29
Allowed (%)	4.71
Outliers (%)	0

Supplementary Table 2. Gene sequences used in this study

NOD1 gene sequence:

ATGGAGGAGCAAGGTCATAGCGAGATGGAGATCATCCCAAGCGAGAGCCATCCTCA
TATCCAGCTGCTGAAGAGCAACCGTGAGCTGCTGGTAACCCATATCCGTAACACCCA
GTGCCTGGTAGACAACCTGCTGAAGAACGACTACTTCAGCGCGGAGGACGCTGAGA
TCGTATGCGCATGTCCTACTCAGCCTGACAAGGTCCGTAAGATCCTGGACCTGGTAC
AAAGCAAGGGTGAGGAGGTCAGCGAGTTCTTCCTGTACCTGCTGCAGCAACTGGCT
GATGCATACGTAGACCTGCGTCCATGGCTGCTGGAAATCGGTTTCAGCCCATCTCTG
CTGACTCAAAGCAAGGTCGTAGTCAACACCGACCCAGTCTCTCGTTACACTCAGCAG
CTGCGTCATCATCTGGGTCGTGACTCTAAGTTCGTGCTGTGTTACGCACAGAAGGAA
GAGCTGCTGCTGGAGGAGATCTACATGGACACCATCATGGAGCTGGTCGGTTTCAGC
AACGAAAGCCTGGGCAGCCTGAACTCTCTGGCATGTCTGCTGGACCATACTACCGGT
ATCCTGAACGAACAGGGTGAAACGATCTTCATCCTGGGCGACGCAGGTGTCGGTAA
GTCTATGCTGCTGCAGCGTCTGCAATCTCTGTGGGCAACTGGTCGTCTGGATGCAGG
TGTTAAGTTCTTTTTTCACTTCCGCTGCCGTATGTTCTCCTGCTTCAAAGAAAGCGAC
CGCCTGTGCCTGCAGGACCTGCTGTTCAAACACTACTGCTACCCGGAACGTGACCCA
GAAGAAGTGTTCGCATTCCTGCTGCGTTTCCCACACGTGGCACTGTTCACTTTCGAC
GGTCTGGACGAACTGCATTCCGACCTGGACCTGTCTCGTGTTCCGGATTCTTCTTGCC
CGTGGGAACCGGCACACCCGCTGGTTCTGCTGGCAAACCTGCTGTCTGGTAAACTGC
TGAAAGGTGCATCTAAACTGCTGACTGCACGTACTGGTATCGAAGTGCCGCGTCAAT
TCCTGCGTAAAAAAGTGCTGCTGCGTGGTTTCTCCCCGTCTCACCTGCGTGCTTATGC
ACGTCGTATGTTCCCGGAACGTGCACTGCAAGATCGTCTGCTGTCTCAACTGGAAGC
TAACCCGAACCTGTGTTCTCTGTGCTCTGTGCCGCTGTTCTGCTGGATCATCTTCCGT
TGCTTCCAGCACTTCCGTGCTGCTTTCGAAGGTTCTCCGCAACTGCCGGATTGTACTA
TGACCCTGACTGACGTGTTCCTGCTGGTGACTGAAGTGCACCTGAACCGTATGCAGC
CGTCTTCTCTGGTGCAACGTAACACTCGTTCCCCGGTTGAAACTCTGCACGCTGGTC
GTGATACTCTGTGCTCTCTGGGTCAGGTTGCTCACCGTGGTATGGAAAAATCCCTGT
TCGTGTTCACCCAGGAAGAAGTGCAGGCTTCTGGTCTGCAGGAACGTGACATGCAG
CTGGGTTTCCTGCGCGCTCTGCCGGAACTGGGTCCTGGTGGTGATCAGCAGTCTTAT
GAATTTTTCCACCTGACTCTGCAGGCTTTCTTCACGGCTTTCTTCCTGGTGCTGGATG
ACCGTGTTGGTACGCAGGAACTGCTGCGTTTTTTCCAGGAATGGATGCCGCCGGCTG
GTGCTGCTACCACCTCTTGTTATCCGCCGTTCCTGCCGTTTCAGTGTCTGCAGGGTTC
TGGCCCGGCTCGTGAAGATCTGTTTAAAAACAAAGACCACTTCCAGTTTACCAACCT
GTTTCTGTGCGGCCTGCTGTCCAAAGCGAAACAGAAACTGCTGCGCCACCTGGTGCC
GGCTGCTGCGCTGCGTCGCAAACGCAAAGCCCTGTGGGCGCACCTGTTTTCTTCTCT
GCGCGGCTACCTGAAATCCCTGCCGCGCGTACAGGTTGAATCCTTCAATCAGGTTCA
GGCGATGCCGACCTTTATTTGGATGCTGCGCTGTATTTACGAAACCCAGAGCCAGAA
AGTTGGCCAGCTGGCGGCGCGCGGCATTTGTGCGAATTATCTGAAACTGACCTACTG
TAATGCGTGTTCCGCCGATTGCTCCGCGCTGAGCTTTGTTCTGCACCACTTTCCGAAA
CGCCTGGCGCTGGATCTGGATAACAATAATCTGAACGATTACGGCGTTCGCGAACTG
CAGCCGTGCTTTTCCCGCCTGACCGTTCTGCGCCTGTCCGTTAATCAGATTACGGATG
GCGGCGTAAAAGTTCTGTCCGAAGAACTGACGAAATACAAAATTGTTACCTACCTG
GGCCTGTATAACAACCAGATCACCGACGTAGGCGCGCGCTATGTAACCAAAATCCT
GGATGAATGCAAAGGCCTGACCCACCTGAAACTGGGCAAAAACAAAATCACCAGCG
AAGGCGGCAAATATCTGGCCCTGGCCGTTAAAAACAGCAAATCCATCTCCGAAGTT

GGCATGTGGGGCAACCAGGTTGGCGATGAAGGCGCGAAAGCGTTTGCCGAAGCCCT
GCGCAACCACCCGAGCCTGACCACCCTGTCCCTGGCCTCCAACGGCATTTCCACCGA
AGGTGGCAAATCCCTGGCCCGCGCGCTGCAGCAGAACACCTCCCTGGAAATTCTGT
GGCTGACCCAGAACGAACTGAACGATGAAGTTGCCGAATCCCTGGCGGAAATGCTG
AAAGTTAACCAGACGCTGAAACACCTGTGGCTGATTCAGAACCAGATTACGGCGAA
AGGCACCGCGCAGCTGGCGGATGCCCTGCAGAGCAACACCGGCATTACCGAAATCT
GCCTGAACGGCAACCTGATTAAACCGGAAGAAGCGAAAGTTTATGAAGATGAAAAA
CGCATCATCTGCTTT

NOD2 Gene sequence:

ATGGGTGAGGAGGGTGGTAGCGCATCTCATGACGAGGAAGAACGTGCTTCTGTACT
GCTGGGTCATTCTCCTGGTTGCGAGATGTGCAGCCAAGAGGCATTCCAGGCTCAGCG
TTCTCAGCTGGTAGAGCTGCTGGTATCCGGTAGCCTGGAGGGTTTCGAGAGCGTCCT
GGATTGGCTGCTGTCTTGGGAGGTTCTGTCTTGGGAAGACTACGAGGGTTTCCACCT
GCTGGGTCAGCCTCTGTCTCACCTGGCTCGTCGTCTGCTGGATACTGTATGGAACAA
GGGTACGTGGGCGTGCCAAAAGCTGATCGCGGCAGCACAAGAGGCTCAAGCAGACT
CCCAAAGCCCAAAGCTGCACGGTTGCTGGGACCCTCATTCTCTGCACCCAGCTCGTG
ACCTGCAATCTCACCGTCCAGCTATCGTACGTCGTCTGCATTCCCACGTCGAGAACA
TGCTGGACCTGGCATGGGAGCGTGGTTTCGTCAGCCAATACGAGTGCGACGAGATC
CGCCTGCCAATCTTCACCCCATCCCAGCGTGCACGTCGCCTGCTGGATCTGGCAACT
GTAAAGGCTAACGGTCTGGCTGCATTCCTGCTGCAGCATGTACAGGAGCTGCCAGTC
CCACTGGCTCTGCCGCTGGAAGCAGCAACTTGTAAGAAGTACATGGCCAAGCTGCG
CACCACTGTGAGCGCACAGTCTCGTTTCCTGTCCACCTACGACGGTGCAGAAACCCT
GTGCCTGGAAGACATCTACACCGAAAACGTGCTGGAAGTCTGGGCCGACGTGGGTA
TGGCGGGTCCTCCTCAGAAGTCTCCGGCAACTCTGGGTCTGGAAGAACTGTTCTCCA
CGCCGGGTCATCTGAACGACGACGCTGACACTGTCCTGGTAGTAGGTGAAGCAGGT
TCCGGTAAATCCACCCTGCTGCAGCGTCTGCATCTGCTGTGGGCAGCAGGTCAAGAC
TTCCAGGAATTTCTGTTCGTGTTCCCGTTCAGCTGCCGCCAGCTGCAGTGCATGGCTA
AACCGCTGAGCGTGCGTACTCTGCTGTTCGAACACTGCTGCTGGCCGGACGTCGGTC
AGGAAGATATCTTTCAGCTGCTGCTGGACCACCCGGACCGTGTTCTGCTGACTTTCG
ACGGTTTCGACGAATTTAAATTTCGCTTCACCGACCGCGAACGTCACTGCAGCCCGA
CCGACCCGACCTCCGTGCAGACTCTGCTGTTTAACCTGCTGCAGGGCAACCTGCTGA
AAAACGCCCGCAAAGTGGTGACCAGCCGTCCGGCAGCTGTGTCTGCATTTCTGCGTA
AATACATCCGTACGGAATTTAACCTGAAAGGCTTCTCCGAACAGGGCATCGAACTGT
ACCTGCGCAAACGTCACCACGAACCGGGCGTGGCGGACCGTCTGATCCGCCTGCTG
CAGGAAACGTCCGCCCTGCACGGTCTGTGCCATCTGCCGGTGTTCTCTTGGATGGTG
TCCAAATGCCACCAGGAACTGCTGCTGCAAGAAGGCGGCAGCCCGAAAACCACGAC
GGACATGTACCTGCTGATCCTGCAGCACTTCCTGCTGCACGCAACCCCGCCGGATTC
TGCATCTCAGGGTCTGGGTCCGTCTCTGCTGCGTGGTCGTCTGCCGACTCTGCTGCAT
CTGGGTCGTCTGGCACTGTGGGGTCTGGGTATGTGTTGTTATGTTTTTCTGCACAGC
AGCTGCAGGCAGCTCAGGTTTCTCCGGATGATATCTCCCTGGGTTTCCTGGTGCGTG
CTAAAGGCGTGGTTCCGGGTTCTACCGCTCCGCTGGAATTTCTGCATATCACCTTCCA
GTGCTTCTTCGCCGCGTTCTACCTGGCGCTGTCTGCTGATGTTCCGCCGGCTCTGCTG
CGTCATCTGTTCAACTGCGGTCGTCCGGGTAATTCTCCGATGGCTCGTCTGCTGCCGA
CTATGTGTATCCAGGCTTCTGAAGGCAAAGATAGCAGCGTTGCCGCCCTGCTGCAGA
AAGCCGAACCGCACAACCTGCAGATCACCGCTGCTTTCCTGGCTGGTCTGCTGTCTC

GTGAACACTGGGGTCTGCTGGCTGAATGCCAAACCTCTGAAAAAGCGCTGCTGCGTC
GTCAGGCTTGCGCTCGTTGGTGTCTGGCTCGTTCTCTGCGTAAACACTTCCACAGCAT
CCCGCCGGCCGCTCCGGGTGAAGCCAAATCTGTTCACGCGATGCCGGGCTTTATTTG
GCTGATTCGTAGCCTGTACGAAATGCAGGAAGAACGCCTGGCGCGTAAAGCGGCGC
GTGGCCTGAATGTTGGCCACCTGAAACTGACTTTCTGCTCCGTTGGCCCGACTGAAT
GCGCGGCGCTGGCGTTTGTTCTGCAGCACCTGCGTCGTCCGGTTGCGCTGCAGCTGG
ATTATAATTCCGTTGGCGATATTGGCGTTGAACAGCTGCTGCCGTGTCTGGGCGTTT
GTAAAGCCCTGTATCTGCGCGATAACAACATCTCCGATCGCGGCATCTGCAAACTGA
TTGAATGCGCCCTGCACTGTGAACAGCTGCAGAAACTGGCGCTGTTCAACAACAAA
CTGACCGATGGCTGTGCGCACTCCATGGCGAAACTGCTGGCGTGTCGCCAGAACTTC
CTGGCCCTGCGCCTGGGCAACAACTATATTACCGCGGCGGGCGCGCAGGTTCTGGCC
GAAGGTCTGCGCGGCAATACCTCCCTGCAGTTTCTGGGCTTTTGGGGCAATCGCGTT
GGCGATGAAGGCGCCCAGGCGCTGGCGGAAGCGCTGGGCGATCACCAGAGCCTGCG
CTGGCTGTCCCTGGTTGGCAACAACATTGGCAGCGTTGGCGCGCAGGCGCTGGCCCT
GATGCTGGCGAAAAACGTTATGCTGGAAGAGCTGTGTCTGGAAGAAAACCACCTGC
AGGATGAAGGCGTTTGTTCCCTGGCGGAAGGCCTGAAAAAAAACTCCAGCCTGAAA
ATTCTGAAACTGAGCAACAACTGTATTACCTATCTGGGCGCGGAAGCTCTGCTGCAG
GCGCTGGAACGCAACGATACCATTCTGGAAGTATGGCTGCGCGGCAACACCTTCTCC
CTGGAAGAAGTAGATAAACTGGGCTGTCGCGATACCCGCCTGCTGCTG

RIP2 gene sequence:

ATGAACGGGGAGGCCATCTGCAGCGCCCTGCCCACCATTCCCTACCACAAACTCGCCGACCTGCGCTACCTGAGCC
GCGGCGCCTCTGGCACTGTGTCGTCCGCCCGCCACGCAGACTGGCGCGTCCAGGTGGCCGTGAAGCACCTGCACA
TCCACACTCCGCTGCTCGACAGTGAAAGAAAGGATGTCTTAAGAGAAGCTGAAATTTTACACAAAGCTAGATTTAG
TTACATTCTTCCAATTTTGGGAATTTGCAATGAGCCTGAATTTTTGGGAATAGTTACTGAATACATGCCAAATGGAT
CATTAAATGAACTCCTACATAGGAAAACTGAATATCCTGATGTTGCTTGGCCATTGAGATTTCGCATCCTGCATGAA
ATTGCCCTTGGTGTAAATTACCTGCACAATATGACTCCTCCTTTACTTCATCATGACTTGAAGACTCAGAATATCTTA
TTGGACAATGAATTTCATGTTAAGATTGCAGATTTTGGTTTATCAAAGTGGCGCATGATGTCCCTCTCACAGTCACG
AAGTAGCAAATCTGCACCAGAAGGAGGGACAATTATCTATATGCCACCTGAAAACTATGAACCTGGACAAAAATC
AAGGGCCAGTATCAAGCACGATATATATAGCTATGCAGTTATCACATGGGAAGTGTTATCCAGAAAACAGCCTTTT
GAAGATGTCACCAATCCTTTGCAGATAATGTATAGTGTGTCACAAGGACATCGACCTGTTATTAATGAAGAAAGTT
TGCCATATGATATACCTCACCGAGCACGTATGATCTCTCTAATAGAAAGTGGATGGGCACAAAATCCAGATGAAAG
ACCATCTTTCTTAAAATGTTTAATAGAACTTGAACCAGTTTTGAGAACATTTGAAGAGATAACTTTTCTTGAAGCTG
TTATTCAGCTAAAGAAAACAAAGTTACAGAGTGTTTCAAGTGCCATTCACCTATGTGACAAGAAGAAAATGGAATT
ATCTCTGAACATACCTGTAAATCATGGTCCACAAGAGGAATCATGTGGATCCTCTCAGCTCCATGAAAATAGTGGT
TCTCCTGAAACTTCAAGGTCCCTGCCAGCTCCTCAAGACAATGATTTTTTATCTAGAAAAGCTCAAGACTGTTATTTT
ATGAAGCTGCATCACTGTCCTGGAAATCACAGTTGGGATAGCACCATTTCTGGATCTCAAAGGGCTGCATTCTGTG
ATCACAAGACCACTCCATGCTCTTCAGCAATAATAAATCCACTCTCAACTGCAGGAAACTCAGAACGTCTGCAGCCT
GGTATAGCCCAGCAGTGGATCCAGAGCAAAAGGGAAGACATTGTGAACCAAATGACAGAAGCCTGCCTTAACCA
GTCGCTAGATGCCCTTCTGTCCAGGGACTTGATCATGAAAGAGGACTATGAACTTGTTAGTACCAAGCCTACAAGG
ACCTCAAAAGTCAGACAATTACTAGACACTACTGACATCCAAGGAGAAGAATTTGCCAAAGTTATAGTACAAAAAT
TGAAAGATAACAAACAAATGGGTCTTCAGCCTTACCCGGAAATACTTGTGGTTTCTAGATCACCATCTTTAAATTTA
CTTCAAAATAAAAGCATG

NLRC4 gene sequence:

ATGAATTTCATAAAGGACAATAGCCGAGCCCTTATTCAAAGAATGGGAATGACTGTTATAAAGCAAATCACAGAT
GACCTATTTGTATGGAATGTTCTGAATCGCGAAGAAGTAAACATCATTTGCTGCGAGAAGGTGGAGCAGGATGCT
GCTAGAGGGATCATTCACATGATTTTGAAAAAGGGTTCAGAGTCCTGTAACCTCTTTCTTAAATCCCTTAAGGAGT
GGAACTATCCTCTATTTCAGGACTTGAATGGACAAAGTCTTTTTCATCAGACATCAGAAGGAGACTTGGACGATTT
GGCTCAGGATTTAAAGGACTTGTACCATACCCCATCTTTTCTGAACTTTTATCCCCTTGGTGAAGATATTGACATTAT
TTTTAACTTGAAAAGCACCTTCACAGAACCTGTCCTGTGGAGGAAGGACCAACACCATCACCGCGTGGAGCAGCT
GACCCTGAATGGCCTCCTGCAGGCTCTTCAGAGCCCCTGCATCATTGAAGGGGAATCTGGCAAAGGCAAGTCCAC
TCTGCTGCAGCGAATTGCCATGCTCTGGGGCTCCGGAAAGTGCAAGGCTCTGACCAAGTTCAAATTCGTCTTCTTC
CTCCGTCTCAGCAGGGCCCAGGGTGGACTTTTTGAAACCCTCTGTGATCAACTCCTGGATATACCTGGCACAATCA
GGAAGCAGACATTCATGGCCATGCTGCTGAAGCTGCGGCAGAGGGTTCTTTTCCTTCTTGATGGCTACAATGAATT
CAAGCCCCAGAACTGCCCAGAAATCGAAGCCCTGATAAAGGAAAACCACCGCTTCAAGAACATGGTCATCGTCAC
CACTACCACTGAGTGCCTGAGGCACATACGGCAGTTTGGTGCCCTGACTGCTGAGGTGGGGGATATGACAGAAG
ACAGCGCCCAGGCTCTCATCCGAGAAGTGCTGATCAAGGAGCTTGCTGAAGGCTTGTTGCTCCAAATTCAGAAATC
CAGGTGCTTGAGGAATCTCATGAAGACCCCTCTCTTTGTGGTCATCACTTGTGCAATCCAGATGGGTGAAAGTGAG
TTCCACTCTCACACACAAACAACGCTGTTCCATACCTTCTATGATCTGTTGATACAGAAAAACAAACACAAACATAA
AGGTGTGGCTGCAAGTGACTTCATTCGGAGCCTGGACCACTGTGGAGACCTAGCTCTGGAGGGTGTGTTCTCCCA
CAAGTTTGATTTCGAACTGCAGGATGTGTCCAGCGTGAATGAGGATGTCCTGCTGACAACTGGGCTCCTCTGTAAA
TATACAGCTCAAAGGTTCAAGCCAAAGTATAAATTCTTTCACAAGTCATTCCAGGAGTACACAGCAGGACGAAGAC
TCAGCAGTTTATTGACGTCTCATGAGCCAGAGGAGGTGACCAAGGGGAATGGTTACTTGCAGAAAATGGTTTCCA
TTTCGGACATTACATCCACTTATAGCAGCCTGCTCCGGTACACCTGTGGGTCATCTGTGGAAGCCACCAGGGCTGT
TATGAAGCACCTCGCAGCAGTGTATCAACACGGCTGCCTTCTCGGACTTTCCATCGCCAAGAGGCCTCTCTGGAGA
CAGGAATCTTTGCAAAGTGTGAAAAACACCACTGAGCAAGAAATTCTGAAAGCCATAAACATCAATTCCTTTGTAG
AGTGTGGCATCCATTTATATCAAGAGAGTACATCCAAATCAGCCCTGAGCCAAGAATTTGAAGCTTTCTTTCAAGG
TAAAAGCTTATATATCAACTCAGGGAACATCCCCGATTACTTATTTGACTTCTTTGAACATTTGCCCAATTGTGCAA
GTGCCCTGGACTTCATTAAACTGGACTTTTATGGGGGAGCTATGGCTTCATGGGAAAAGGCTGCAGAAGACACAG
GTGGAATCCACATGGAAGAGGCCCCAGAAACCTACATTCCCAGCAGGGCTGTATCTTTGTTCTTCAACTGGAAGCA
GGAATTCAGGACTCTGGAGGTCACACTCCGGGATTTCAGCAAGTTGAATAAGCAAGATATCAGATATCTGGGGAA
AATATTCAGCTCTGCCACAAGCCTCAGGCTGCAAATAAAGAGATGTGCTGGTGTGGCTGGAAGCCTCAGTTTGGT
CCTCAGCACCTGTAAGAACATTTATTCTCTCATGGTGGAAGCCAGTCCCCTCACCATAGAAGATGAGAGGCACATC
ACATCTGTAACAAACCTGAAAACCTTGAGTATTCATGACCTACAGAATCAACGGCTGCCGGGTGGTCTGACTGACA
GCTTGGGTAACTTGAAGAACCTTACAAAGCTCATAATGGATAACATAAAGATGAATGAAGAAGATGCTATAAAAC
TAGCTGAAGGCCTGAAAAACCTGAAGAAGATGTGTTTATTTCATTTGACCCACTTGTCTGACATTGGAGAGGGAAT
GGATTACATAGTCAAGTCTCTGTCAAGTGAACCCTGTGACCTTGAAGAAATTCAATTAGTCTCCTGCTGCTTGTCTG
CAAATGCAGTGAAAATCCTAGCTCAGAATCTTCACAATTTGGTCAAACTGAGCATTCTTGATTTATCAGAAAATTAC
CTGGAAAAAGATGGAAATGAAGCTCTTCATGAACTGATCGACAGGATGAACGTGCTAGAACAGCTCACCGCACTG
ATGCTGCCCTGGGGCTGTGACGTGCAAGGCAGCCTGAGCAGCCTGTTGAAACATTTGGAGGAGGTCCCACAACTC
GTCAAGCTTGGGTTGAAAAACTGGAGACTCACAGATACAGAGATTAGAATTTTAGGTGCATTTTTTGGAAAGAAC
CCTCTGAAAAACTTCCAGCAGTTGAATTTGGCGGGAAATCGTGTGAGCAGTGATGGATGGCTTGCCTTCATGGGT
GTATTTGAGAATCTTAAGCAATTAGTGTTTTTTGACTTTAGTACTAAAGAATTTCTACCTGATCCAGCATTAGTCAG
AAAACTTAGCCAAGTGTTATCCAAGTTAACTTTTCTGCAAGAAGCTAGGCTTGTTGGGTGGCAATTTGATGATGAT
GATCTCAGTGTTATTACAGGTGCTTTTAAACTAGTAACTGCTTAA

Supplementary Table 3. Primer sequences

Vector cloning					
gene	domain	Vector	Restriction site	Forward primer (5'-3')	reverse primer (5'-3')
NOD1	Full length	pcDNA3.1	BamHI/XbaI	ATAAGGATCCATGTACCCATACGATGTTCCAGATTACGCTGAGGAGCAAGGTC	ATAATCTAGATCAAAAGCAGATGATGCGTTTTTCATCTTCATAAACTTTCGC
NOD1	CARD	pcNDA3.1	Kpn1/BamHI	ATAAGGTACCATGGAGGAGCAAGGTCATAGCGAG	ATAAGGATCCGCTGAAACCGATTTCCAGCAGCC
NOD1	CARD	pET47	XmaI/EcoRI	ATAACCCGGGGAGAGCCATCCTCATATCCAGCTGCTGAAGAGC	ATAAGAATTCGCTGAAACCGATTTCCAGCAGCCATGGAC
NOD2	CARD	pet47	XmaI/EcoRI	ATAACCCGGGATGGGTGAGGAGGGTGGTAGCGC	ATAAGAATTCCTACCATCTTACAAGTTGCTGCTTCCAGCG
NOD2	Full length	pcDNA3.1	BamHI/XbaI	ATAAGGATCCATGGGTGAGGAGGGTGGTAGCGCATCTCAT	ATAATCTGAGCAGCAGCAGGCGGGTATCGCGACAGCCCAGTTTATCT
NOD2	CARD	pcDNA3.1	HindIII/XhoI	ATAAAAGCTTATGTGCAGCCAAGAGGCATTCCAG	ATAACTCGAGGGGTCCCTGAAAGAGGACTTCCAGCGGCAG
NOD1	CARD	pET47	EcoRI/XhoI	ATAAGAATTCGAGAGCCATCCTCATATCCAGCTGCTGAAGAGC	ATAACTCGAGGCTGAAACCGATTTCCAGCAGCCATGGAC
NOD2	CARD	pET47	EcoRI/XhoI	ATAAGAATTCGATGTGCAGCCAAGAGGCATTCCAGGCTCAGCGTTC	ATAACTCGAGGGGTCCCTGAAAGAGGACTTCCAGCGGCAG
RIP2	CARD	Vsnap-tag T7(2)	XhoI/NotI	ATAACTCGAGCAGCCTGGTATAGCCCAGCAG	ATAAGCGGCCGCCTAATGGTGATGATGGTGGTGCATGCTTTTATTTTGAAGTAAATTTAAAG
RIP2	Full length	pcDNA3.1	HindIII/XhoI	ATAAAAGCTTATGGACTACAAGGACGATGACGATAAGAACGGGGAGGCCATCTG	ATAACTCGAGTCACATGCTTTTATTTTGAAGTAAATTTAAAG
RIP2	CARD	pcDNA3.1	XhoI/XbaI	GATAACTCGAGATAGCCCAGCAGTGGATCCAGAGC	GATAATCTAGACTATGATCTAGAAACCACAAGTATTTCCGGG

MBP	Full length	pcDNA3.1	HindIII/BamHI	ATAAAAGCTTATGGACTACAAGGACGATGACGATAAGAAAATCGAAGAAGGTAAACTGG	ATAAGGATCCAGTCTGCGCGTCTTTCAGGGCTTCATG
RIP2	Kinase	pcDNA3.1	XmaI/EcoRI	ATAACCCGGGAACGGGGAGGCCATCTGCAGCGCC	ATAACTCGAGACTCTGTAACTTTGTTTTCTTTAGCTG
NLRC4	CARD	pcDNA3.1	EcoRI/NotI	ATAAGAATTCATGAATTTCATAAAGGACAATAGCCGAGCC	ATAAGCGGCCGCTTATTCTGATGTCTGATGAAAAAGACTTTGTCC
NLRC4	Full length	pcDNA3.1	Kpn1/XhoI	ATAAGGTACCATGGACTACAAGGACGATGACGATAAGAATTTCATAAAGGACAATAGCCG	ATAACTCGAGTTAAGCAGTTACTAGTTTAAAAGCACCTGTAATAACACTG
MAVS	CARD	pcDNA3.1	HindIII/XhoI	ATAAAAGCTTATGCCGTTTGCTGAAGACAAGACCTATAAGTATATC	ATAACTCGAGCGAGGTCCGAGGCTGGTAGCTCTGGTAGAC

Mutagenesis				
gene		mutation		primer
NLRC4		E36R		CCTATTTGTATGGAATGTTCTGAATCGCAGGGAAGTAAACATCATTTGCTGCGAGAAG
NOD1		ED53AA		GAACGACTACTTCAGCGCGGCGGCCGCTGAGATCGTATGCGCATG
NOD1		RK69AA		CTACTCAGCCTGACAAGGTC GCTGCG ATCCTGGACCTGGTACAAAGC
NOD1		K67A		GCATGTCCTACTCAGCCTGAC GCG GTCCGTAAGATCCTGGACCTG
NOD1		E28A		ATCCAGCTGCTGAAGAGCAACCGTGCCCTGCTGGTAACCCATATCCGTAAC
NOD2		ED69AA		CTTGGGAGGTTCTGTCTTGG GCAGCC TACGAGGGTTTCCACCTGC
NOD2		RR143AA		CTCACCGTCCAGCTATCGTA GCTGCT CTGCATTCCCACGTCGAG
RIP2		E445R		AGCCCAGCAGTGGATCCAGAGCAAAAGGAGAGACATTGTGAACCAAATGACAGAAGCCTG
RIP2		R444E		AGCCCAGCAGTGGATCCAGAGCAAAGAGGAAGACATTGTGAACCAAATGACAGAAGCCTG
RIP2		D492K		GGACCTCAAAAGTCAGACAATTACTAAAAACTACTGACATCCAAGGAGAAGAATTTGCC
RIP2		D461R		ATGACAGAAGCCTGCCTTAACCAGTCGCTAAGGGCCCTTCTGTCCAGGGACTTGATCATG
RIP2		Y474R		CCAGGGACTTGATCATGAAAGAGGACAGGGAACTTGTTAGTACCAAGCCTACAAGGACC
RIP2		Q458E		AACCAAATGACAGAAGCCTGCCTTAACGAGTCGCTAGATGCCCTTCTGTCCAGGGACTTG
RIP2		E472R		CCTTCTGTCCAGGGACTTGATCATGAAAAGGGACTATGAACTTGTTAGTACCAAGCCTAC

RIP2		Q497E		TCAGACAATTACTAGACACTACTGACATCGAGGGAGAAGAATTT GCCAAAGTTATAGTAC
RIP2		K513E		GTACAAAAATTGAAAGATAACGAGCAAATGGGTCTTCAGCCTTA CCCGGAAATACTTG
RIP2		N449D		ATCCAGAGCAAAAGGGAAGACATTGTGGACCAAATGACAGAAG CCTGCCTTAACCAGTCG
RIP2		K471D		GTCCAGGGACTTGATCATG GACGAGGACTATGAACTTGTTAG
RIP2		E475R		CAGGGACTTGATCATGAAAGAGGACTATCGCCTTGTTAGTACCA AGCCTACAAGGACCTC
RIP2		R488E		TACCAAGCCTACAAGGACCTCAAAAGTCGAGCAATTACTAGACA CTACTGACATCCAAGG
RIP2		R483D		CTTGTTAGTACCAAGCCTACA GAC ACCTCAAAAGTCAGACAATTA
RIP2		D495R		AGACAATTACTAGACACTACT AGG ATCCAAGGAGAAGAATTTGCC

Supplementary Table 4. List of Commercial Antibodies and Reagents

Item	brand	RRID	catalog no	lot no
Anti-RIP2 antibody	Abcam	AB_2178250	ab8427	GR7926
RIP2 (D10B11) Rabbit mAb	Cell Signaling Technology	AB_2716277	4142S	
Phospho-RIP2 (Ser176) (E1I9J) Rabbit mAb	Cell Signaling Technology		14397S	
Anti-NOD1 Antibody	Cell Signaling Technology	AB_10695865	3545S	
Anti-NOD2 Antibody (2D9)	Santa Cruz Biotechnology	AB_781804	sc-56168	
Anti-Ubiquitin antibody	Abcam	AB_306069	ab7780	GR31967083
Monoclonal ANTI-FLAG® M2-Peroxidase (HRP)	Sigma-Aldrich	AB_439702	A8592	SLBK9652V
Monoclonal Anti-β actin	Sigma-Aldrich	AB_476697	A2228	066M4860V
Goat Anti-Rabbit IgG H&L (HRP)	Abcam	AB_955447	ab6721	GR313248
Rabbit Anti-Mouse IgG H&L (HRP)	Abcam	AB_10680920	ab97046	GR315070
Dual-Luciferase Reporter Assay System 10-Pack	Promega		E1960	0000180543(1)
Fugene HD Transfection reagent	Promega		E2311	0000259642(1)
Passive Lysis 5x Buffer	Promega		E1941	0000181473(1)
Neon™ Transfection System 100 µL Kit	Invitrogen		MPK10096	2K12822
Nativepage 3-12% Gels, 15 Well 10 per pack	Invitrogen		BN1003BOX	17060660
Alexa Fluor™ 647 Phalloidin	Invitrogen		A22287	
DMEM/HIGH Glucose, with L-Glutamine & Sodium Pyruvate	Gibco		11995-065	
Fetal bovine serum	Gibco		10270-106	
PBS (1X) Liquid, w/o CaCl & MgCl, pH=7.2	Gibco		20012-027	
Trypsin (0.25%, 1x, with 1mM EDTA.4Na)	Gibco		25200-056	
Poly-L-lysine solution	Sigma-Aldrich		P4707	
MagVigen™- anti-Flag,mouse	Nvigen		K51007	

Blocking grade blocker	Biorad		1706404	
Bovine Serum Albumin Lyophilised pH~7 - 100g	Biowest		P6154-100 GR	
Plasmid Midi Prep Kit	Qiagen		12145	151028802
Monarch® Nucleic Acid Purification Kits	New England BioLabs Inc		T1010L	61705
Kapa Pfu polymerase	KAPA Biosystem		KK2102A	
Kapa High Fidelity DNA polymerase	KAPA Biosystem		KK2101	
Q5 High Fidelity DNA polymerase	New England BioLabs Inc		M0491L	
Taq DNA Polymerase with ThermoPol® Buffer	New England BioLabs Inc		M0267L	
SNAP-Surface® 649	New England BioLabs Inc		S9159S	
SNAP-Surface® Alexa Fluor® 488	New England BioLabs Inc		S9129S	
DAPI	Sigma-Aldrich		D9542	
Paraformaldehyde	Sigma-Aldrich		P6148	
Tween 20, EIA grade, 100ML	Biorad		1706531	
Tris base	Affymetrix		75825	4302149
NaCl	Merck		1.06404.5000	K48328204650
EDTA, Disodium salt dihydrate. Electrophoresis purity	Biorad		1610729	
Triton X-100	Biorad		1610407	
UltraPure™ Sodium Dodecyl Sulfate (SDS)	Invitrogen		15525-017	16H235305
Sodium deoxycholate	Sigma-Aldrich		D6750	086K0045V
WesternBright Sirius,femtogram HRP Substrate	Advansta		K-12043-D20	
Glycine	Sigma-Aldrich		G7126	011M0114V

Supplementary Table 5 Statistical data of chart plots, with two decimal numbers.

Figure 1

	Firefly/Renilla						
	siRNA #1#2						
	**NOD1-FL**		**NOD1-CARD**		**NOD2-FL**		
	**50ng**	**200ng**	**50ng**	**200ng**	**50ng**	**200ng**	
avg	6.35	5.17	4.65	4.83	6.39	4.39	
std	0.11	0.06	0.15	0.12	0.25	0.13	
	**NOD2-CARD**		**RIP2-FL**		**RIP2-CARD**		**pFlag**
	**50ng**	**200ng**	**50ng**	**200ng**	**50ng**	**200ng**	**200ng**
avg	5.33	5.80	15.46	37.78	0.47	0.49	0.37
std	0.22	0.22	0.33	0.80	0.00	0.01	0.01

	avg	std
**NOD1 50ng**	65.01	1.03
**NOD1 200ng**	210.60	8.66
**NOD2 50ng**	73.70	0.85
**NOD2 200ng**	212.14	5.91
**NOD1-CARD 50ng**	42.74	0.91
**NOD1-CARD 200ng**	225.95	4.62
**NOD2-CARD 50ng**	45.03	0.20
**NOD2-CARD 200ng**	175.43	1.64
**RIP2 50ng**	6.10	0.15
**RIP2 200ng**	135.86	4.20
**RIP2-CARD 50ng**	2.04	0.03
**RIP2-CARD 200ng**	12.01	0.29
**pFlag**	0.81	0.02

Figure 2

	**NLRC4-FL**			**RIP2 kinase NLRC4 CARD**		
	**100ng**	**200ng**	**500ng**	**100ng**	**200ng**	**500ng**
AVG	0.96	1.12	2.20	45.24	86.74	211.49
STD	0.02	0.02	0.09	2.53	1.17	2.63

	**RIP2 kinase NLRC4 CARD E36R**			**RIP2**	**K513E**	**pFlag**
	**100ng**	**200ng**	**500ng**	**200ng**	**200ng**	**500ng**
AVG	0.66	1.12	1.72	49.25	0.63	0.41
STD	0.01	0.01	0.08	0.54	0.03	0.03

Figure 3

Firefly/Renilla				
**RIP2 protein**				
**13aa 529**	**13aa 540**	**13aa540 K513E**	**Flag-IS**	**pFlag**
avg				
33.80	10.15	0.59	39.49	0.35
std				
6.80	0.03	0.01	0.20	0.02

Figure 6

	AVG	STD
**pFlag**	0.77	0.02
**RIP2**	128.67	1.81
**N449D**	18.43	1.10
**N449R&D495R**	36.88	0.10
**N449R**	282.17	8.04
**D495R**	181.16	7.50
**N449D&D495R**	237.33	0.77
	AVG/PFLAG	STD/PFLAG
**E445R**	10.90	0.14
**Y474R**	3.64	0.21
**Q458E**	7.09	0.17
**E472R**	1.91	0.00
**R483D**	2.13	0.05
**pFlag**	1.00	0.08
**RIP2**	110.38	2.39
**R488E**	2.06	0.04
**R444E**	3.79	0.11
**N449D**	18.09	1.14
**D461R**	2.61	0.09
**D492K**	1.93	0.04
**R488E**	2.67	0.03
**K471D**	1.56	0.08

**E475R**	1.90	0.03
**Q497E**	10.02	0.33
**K513E**	18.35	0.96
	AVG/PFLAG	STD/PFLAG
**E445R**	10.90	0.14
**Q458E**	7.09	0.17
**Q458E&E445R**	2.57	0.06
**RIP2**	110.38	2.39
**pFlag**	1.00	0.08

Figure 7

	**iE-DAP (ug/mL)**				
	**1**	**10**	**100**	**400**	**0**
avg	1.42	3.90	5.60	6.34	1.00
std	0.73	0.79	0.86	1.02	0.00

Supplementary Figure 1

	siRNA #3#4						
	Firefly/Renilla						
	**NOD1-FL**		**NOD2-FL**		**RIP2-FL**		
	**50ng**	**200ng**	**50ng**	**200ng**	**50ng**	**200ng**	
avg	5.26	3.15	6.89	5.57	25.93	68.05	
std	0.29	2.84	0.22	0.03	0.92	3.18	
	**NOD1-CARD**		**NOD2-CARD**		**RIP2-CARD**		**pFlag**
	**50ng**	**200ng**	**50ng**	**200ng**	**50ng**	**200ng**	**200ng**
avg	3.68	4.68	4.42	4.00	0.46	0.54	0.34
std	0.01	0.19	0.19	0.21	0.02	0.03	0.01
